# Resolving the immune landscape of human prostate at a single-cell level in health and cancer

**DOI:** 10.1016/j.celrep.2021.110132

**Published:** 2021-12-21

**Authors:** Zewen Kelvin Tuong, Kevin W. Loudon, Brendan Berry, Nathan Richoz, Julia Jones, Xiao Tan, Quan Nguyen, Anne George, Satoshi Hori, Sarah Field, Andy G. Lynch, Katarzyna Kania, Paul Coupland, Anne Babbage, Richard Grenfell, Tristan Barrett, Anne Y. Warren, Vincent Gnanapragasam, Charlie Massie, Menna R. Clatworthy

**Affiliations:** 1 Molecular Immunity Unit, Department of Medicine, University of Cambridge, Cambridge, UK; 2 Cellular Genetics, Wellcome Sanger Institute, Hinxton, UK; 3 Department of Oncology, Cambridge University Hospitals NHS Foundation Trust, Cambridge, UK; 4 Department of Urology, Cambridge University Hospitals NHS Foundation Trust, Cambridge, UK; 5 CRUK Cambridge Institute, Cambridge, UK; 6 Division of Genetics and Genomics, Institute for Molecular Bioscience, The University of Queensland, Brisbane, Australia; 7 Early Detection Programme, CRUK Cambridge Centre, Cambridge, UK; 8 Academic Urology Group, Department of Surgery, University of Cambridge, Cambridge, UK; 9 School of Mathematics and Statistics/School of Medicine, University of St Andrews, St Andrews, UK; 10 Department of Radiology, University of Cambridge, Cambridge, UK; 11 Cambridge University Hospitals NHS Foundation Trust, Cambridge, UK; 12 Cambridge Urology Translational Research and Clinical Trials, Cambridge Biomedical Campus, Cambridge, UK; 13 NIHR Cambridge Biomedical Research Centre, Cambridge, UK; 14 Cambridge Institute of Therapeutic Immunology & Infectious Diseases, Cambridge, UK

**Keywords:** single-cell RNA sequencing, macrophage, zinc, immune landscape, human prostate, prostate cancer

## Abstract

The prostate gland produces prostatic fluid, high in zinc and citrate and essential for the maintenance of spermatozoa. Prostate cancer is a common condition with limited treatment efficacy in castration-resistant metastatic disease, including with immune checkpoint inhibitors. Using single-cell RNA-sequencing to perform an unbiased assessment of the cellular landscape of human prostate, we identify a subset of tumor-enriched androgen receptor-negative luminal epithelial cells with increased expression of cancer-associated genes. We also find a variety of innate and adaptive immune cells in normal prostate that were transcriptionally perturbed in prostate cancer. An exception is a prostate-specific, zinc transporter-expressing macrophage population (MAC-MT) that contributes to tissue zinc accumulation in homeostasis but shows enhanced inflammatory gene expression in tumors, including T cell-recruiting chemokines. Remarkably, enrichment of the MAC-MT signature in cancer biopsies is associated with improved disease-free survival, suggesting beneficial antitumor functions.

## Highlights

• An immune cell atlas of healthy human prostate and prostate cancer
• Low androgen receptor-expressing luminal epithelial cell present in prostate cancer
• Metallothionein-expressing macrophage subset (MAC-MT) regulates prostate zinc
• MAC-MT gene signature in prostate cancer associated with better outcomes

## Graphical abstract

**Figure:**
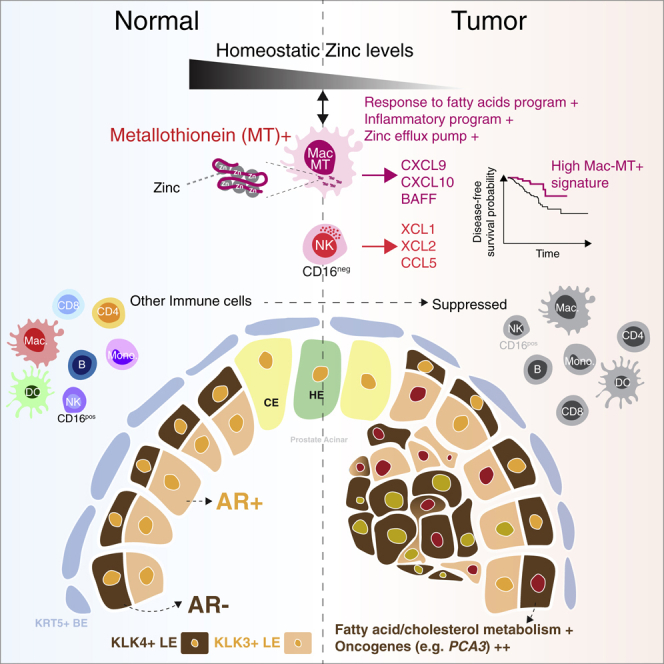

## Teaser

Tuong et al. generated a single-cell transcriptomic map of the human prostate immune landscape in health and show how this is perturbed in cancer. They identify a prostate-specific macrophage population that helps maintain tissue zinc and is associated with better outcomes in cancer.

## Introduction

The prostate gland is critical for human reproduction, generating prostatic fluid that is high in zinc and citrate. This forms an essential component of seminal fluid that is required for the maintenance of spermatozoa (Costello and Franklin, 2016). Prostatic acini are comprised of an outer basal cell layerand inner layers of secretory luminal epithelial (LE) cells; as well as neuroendocrine, stromal, immune, endothelial, and nerve cells (Shen and Abate-Shen, 2010, Henry et al., 2018). Prostate cancer is a major cause of cancer-related mortality and morbidity in men (Ferlay et al., 2015) and is characterized by a reduction in zinc and citrate concentration in both glandular tissue and prostatic fluid (Costello and Franklin, 2016). Cancer cells display a luminal phenotype but the cellular origin of prostate cancer is debated with lineage tracing studies in mice indicating that they may arise from both basal and luminal cells (Choi et al., 2012, Wang et al., 2013, Wang et al., 2014).

There is limited information on the nature and composition of the tissue-resident immune cell compartment in the healthy human prostate (Shen and Abate-Shen, 2010, Henry et al., 2018). However, several tumor-associated immune cell subsets have been reported, including T and B lymphocytes, regulatory T cells, monocytes, macrophages, dendritic cells (DCs), and natural killer (NK) cells (Hussein et al., 2009, Solinas et al., 2017), some of which correlate with a worse prognosis—for example, regulatory T cells (Davidsson et al., 2013) and CD163-positive M2 macrophages (Erlandsson et al., 2019).

Prostate cancer is classified according to the ISUP Grade Group system (Epstein et al., 2016) and the Gleason Grading System, based on the extent to which tissue architecture and cellular morphology are disrupted, with higher scores associated with more aggressive tumor growth and worse outcomes. While primary localized disease has a generally good prognosis, men with locally advanced and metastatic disease have much worse 10-year survival rates (Gnanapragasam et al., 2016). Androgen deprivation therapy is the mainstay of treatment for *de novo* metastatic prostate cancer, but a proportion of patients progress to castration-resistant disease, although the mechanisms underpinning this are unclear (Watson et al., 2015). Immune checkpoint blockade with antibodies against cytotoxic-T-lymphocyte-associated protein 4 (CTLA4) or programmed cell death 1 (PD-1)/PD-1 ligand 1 (PD-L1) has been used in these patients, but results have largely been disappointing (Kwon et al., 2014, Beer et al., 2017), consistent with reports that less than a third of tumors show evidence of PD1 or PDL1 expression (Haffner et al., 2018). Therefore, there is an urgent need to better understand tissue immunity in the healthy prostate and the nature of its perturbation in prostate cancer to inform future therapeutic strategies. Here, we applied single-cell RNA sequencing (scRNaseq) to paired human prostate biopsies collected at the time of cancer diagnosis, to comprehensively profile the cellular landscape of normal human prostate and to determine how this becomes disrupted in cancer. We validated our findings using flow cytometry, immunofluorescence microscopy, spatial transcriptomics; and by cross-species studies of mouse prostate specimens.

## Results

### Single-cell landscape of healthy prostate and prostate cancer

**Figure 1:**
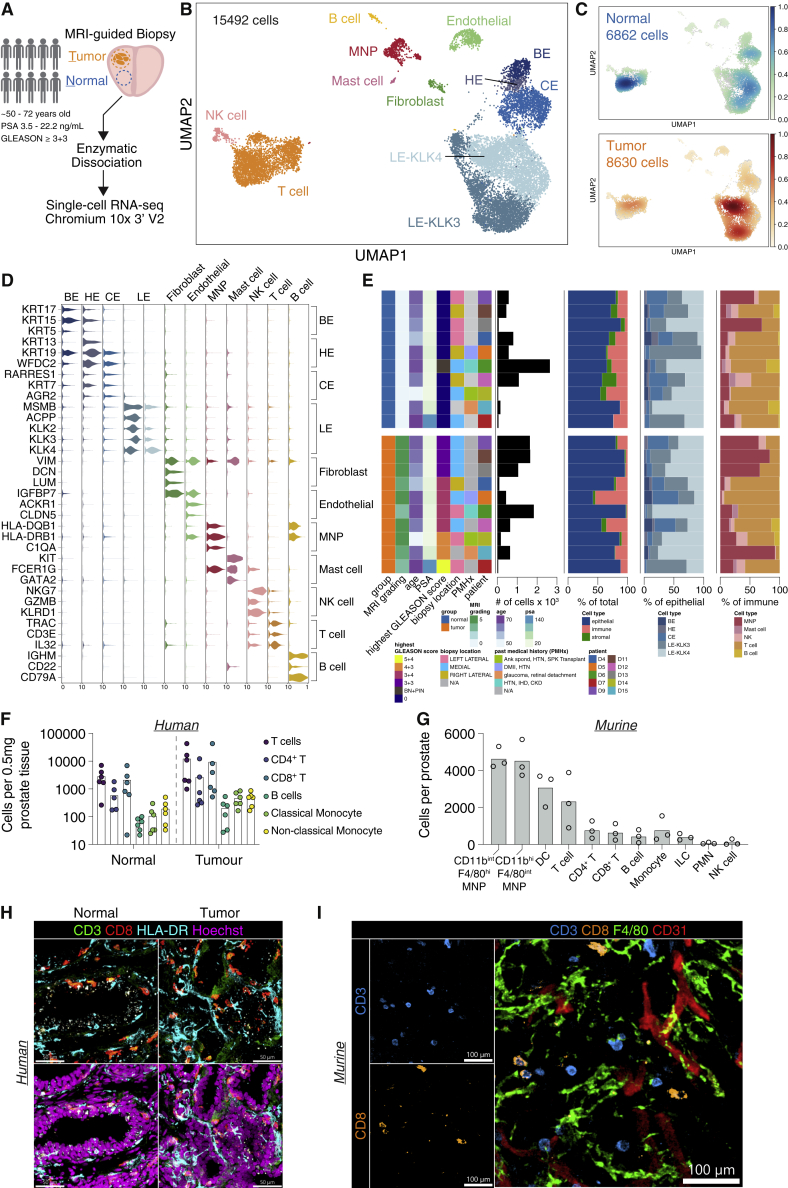
Single-cell RNA sequencing reveals immune and epithelial cell heterogeneity in paired normal-cancer samples (A) Schematic describing experimental set-up for 10x genomic single-cell RNaseq of matched tumor-normal prostate samples from n = 10 patients. (B) UMAP of 15,492 cells post-QC from all prostate samples. (C) UMAP of embedding density of source of samples (normal—green to blue gradient, top; tumor—orange to red gradient, bottom). (D) Violin plot of canonical marker genes for each cell types found in prostate. Gene expression values per cell are standardized to a range from 0 to 10. (E) Patient demographics displayed as a color-coded heatmap and stacked bar charts of single-cell cell type proportions. (F) Quantification of absolute cells counts by flow cytometry per 0.5mg of normal and malignant human prostatic tissue for the indicated immune subsets. Data are a combination of n = 6 donors with each dot representing an individual donor. (G) Quantification of absolute cells counts by flow cytometry per murine prostate lobe for the indicated immune subsets. Each dot represents an individual mouse (n = 3 biological replicates). Abbreviations: MNP—mononuclear phagocyte; DC—dendritic cell; ILC—innate-like lymphoid cell; PMN—polymorphonuclear; NK natural killer (H) Confocal imaging of CD3, CD4 and CD8 in human normal and tumor prostate. Scale bars, 50 μm. (I) Confocal imaging of CD3, CD8, F4/80 and CD31 in normal murine prostate. Scale bars, 100 μm. See also Figure S1 and Table S1.

We performed scRNaseq on paired cancer biopsy and adjacent normal prostate tissue in n = 10 subjects aged 50–72 years of age (Figure 1A, Table S1) generating data on 15,492 cells post-QC. We identified 14 cell clusters (Figure 1B), all of which contained cells from both normal and cancer biopsies (Figures 1C and S1A). This included immune cells, endothelial cells, fibroblasts, as well as several epithelial cell subtypes (Figure 1B), that were annotated based on canonical marker expression and comparison with a previously published scRNA seq dataset of young, healthy human prostate tissue (Henry et al., 2018) (Figures 1D and S1B).

Immune cell clusters included mononuclear phagocytes (MNPs), mast cells, NK cells, B cells, and T cells (Figure 1D) that were present in both normal and cancer samples with similar frequency (Figure 1E). We used flow cytometric analysis and confocal imaging to validate the presence of the major immune cell subsets in normal prostate tissue in human, and performed a cross-species comparison in mice, using a CD45 antibody administered intravenously premortem to label intravascular cells and confirm bona fide tissue residency (Figures 1F, 1G, 1H, 1I, S1C, S1D, and S1E). Together, these data show that the healthy prostate has a rich immune landscape dominated by T cells and MNPs, and that these cells persist in prostate cancer.

### Distinct subset of luminal epithelial cells enriched in cancer

**Figure 2:**
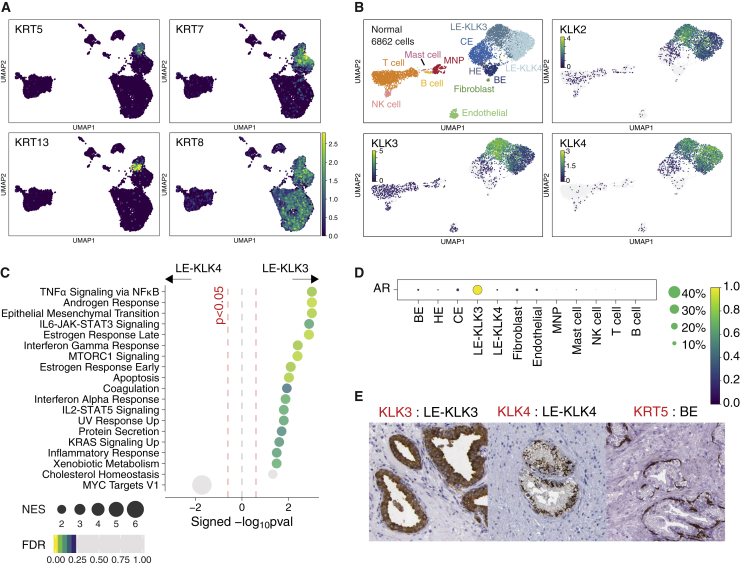
Androgen-receptor-negative prostate luminal epithelial cell type (A) UMAP expression plot of keratin genes in prostate cells. Increasing color gradient from purple, blue, green to yellow corresponds to increasing (standardized) expression value. (B) UMAP of normal prostate sample cells. Expression of kallikrein genes marking luminal epithelial cell types, including luminal cell type, is presented as a heatmap where cells with no expression (0 expression) are colored gray and increasing expression is colored according to increasing gradient from purple, blue, green to yellow. (C) Pre-ranked GSEA of hallmark gene sets between normal KLK3+ versus KLK4+ LE clusters. Pathways with FDR < 0.25 are colored from purple, blue, green to yellow according to decreasing FDR value. Grey circles indicate pathways that attained p < 0.05 and FDR > 0.25. Size of circles indicate the significance (signed -log_10_(p value)). (D) Mean expression dot plot of gene encoding androgen receptor (AR). Expression values are scaled from 0 to 1. Size of circles indicate percentage of cells expressing the gene and increasing color gradient from purple, blue, green to yellow corresponds to increasing (standardized) expression value. (E) Immunohistochemistry images of KLK3, KLK4 and KRT5 in prostate tissue. Images are sourced from the Human protein atlas (https://www.proteinatlas.org). See also Figures S2–S3.

Among nonimmune cell clusters, we identified basal, hillock, and club cell clusters, as noted in previous single cell analyses of human prostate (Henry et al., 2018, Karthaus et al., 2020), but in contrast to published data, we found two distinct clusters of cytokeratin-8+ luminal epithelial (LE) cells, rather than a single LE cell population (Figure 2A). The two LE cell populations were present in normal prostate and prostate cancer samples and were transcriptionally distinct with one cluster expressing high levels of KLK3 (encoding kallikrein related peptidase 3—also known as prostate-specific antigen [PSA]), KLK2, and KLK4 (annotated as LE-KLK3)—and the second cluster expressing high levels of KLK4 and little KLK3 (annotated as LE-KLK4) (Figure 2B). Cells with a high degree of transcriptional similarity to LE-KLK4 were confirmed to be present in the previously published healthy prostate dataset generated from three organs donors aged 18–31 years of age (Henry et al., 2018; Figure S2A), but the limited number of LE-KLK4 cells in this dataset did not enable their identification as a distinct subset in the previously published analysis (Henry et al., 2018). Similarly, examination of previously published prostate cancer single-cell datasets (Karthaus et al., 2020, Chen et al., 2021, Crowley et al., 2020) also identified cells with high transcriptional similarity to LE-KLK3 and LE-KLK4 (Figures S2B–S2D). LE-KLK3 demonstrated enrichment for several immune pathways, including ‘*TNFa via NFKB signaling’*, ‘*IL6-JAK-STAT3 signaling’* ‘*interferon gamma response’*, as well as ‘*androgen and estrogen response’*, with LE-KLK4 showing some enrichment for ‘*Myc target’* genes suggestive of proliferative activity (Figure 2C). Furthermore, a subset of LE-KLK4 cells enriched for a prostate tumor-associated proliferation ‘*Polaris*’ signature (Figure S3A) and included cells with a G2/M cell-cycle profile (Figure S1A). In keeping with the high expression of ‘*androgen response’* pathway genes in LE-KLK3, the gene encoding the androgen receptor (AR) was also highly expressed in this cluster, with little AR expression in LE-KLK4 (Figure 2D). Protein atlas data demonstrated the presence of KLK4+ cells between KLK5+ basal cells and KLK3+ cells adjacent to the lumen (Figure 2E). Colocalization of LE-KLK3 and LE-KLK4 cells was also confirmed in a previously published spatial transcriptomics dataset (Berglund et al., 2018; Figure S3B). The closer spatial proximity of LE-KLK3 to the lumen compared with LE-KLK4 is consistent with the transcriptional enrichment in immune defense genes observed in LE-KLK3, as these cells present an interface with the external environment and potential pathogen challenge.

### Zinc transporter-expressing prostate-specific macrophage population

**Figure 3:**
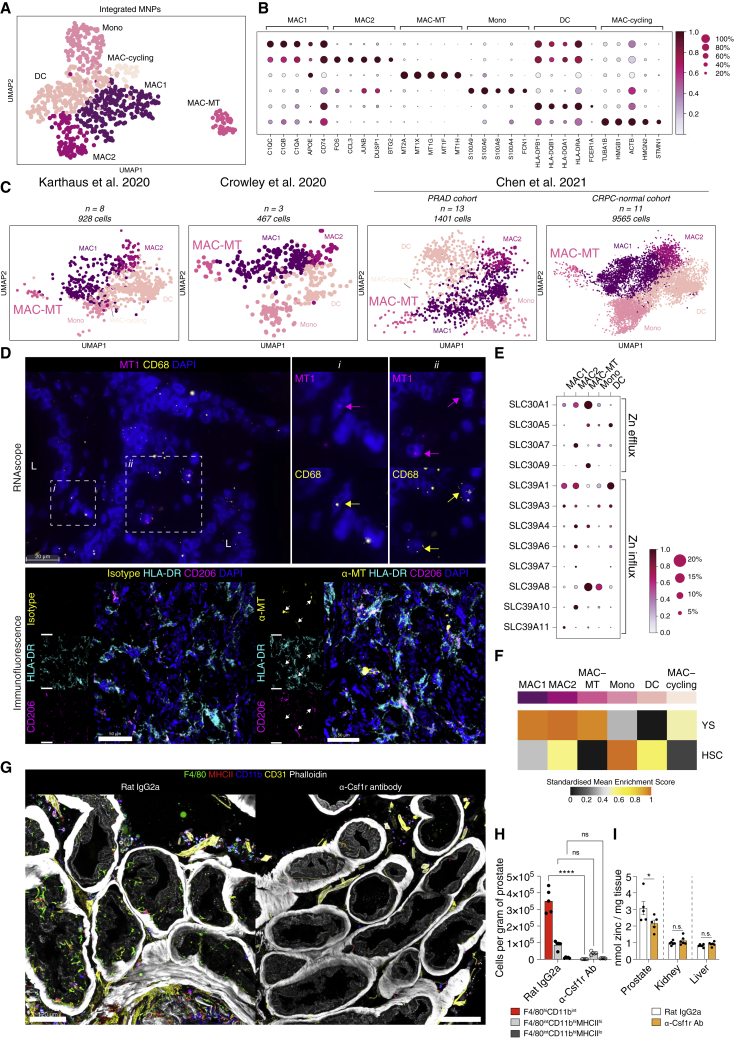
Immune landscape of the prostate includes a prostate-specific macrophage subset enriched in metallothionein transcripts (A) UMAP of 793 cells in myeloid compartment after integration of myeloid/MNP cells from n = 10 patients with Henry et al. myeloid/MNP cells. (B) Mean expression dot plot of top five significant marker genes for each myeloid cluster. Marker genes were identified using Wilcoxon rank sum test and p adj < 0.05 was considered statistically significant. Size of circles indicate percentage of cells expressing the gene and increasing color gradient from white to red corresponds to increasing expression value. (C) UMAP plot of predicted MNP clusters in prostate cancer single cell data from (Karthaus et al., 2020, Chen et al., 2021, Crowley et al., 2020). (D) (top) Representative RNAscope images of probes targeting MT1 family genes (magenta) and CD68 (yellow). ‘L’ indicates lumen. Arrows point to single cells that are marked by both probes in sub-panels i and ii. Scale bar, 20 μm. (bottom) Representative immunofluorescence microscopy images of a human prostate section labeled for metallothionein (α-MT)/isotype control (yellow), HLA-DR (cyan), CD206 (purple) and DAPI (blue). White arrows point to structure displaying colocalization of α-MT with HLA-DR and/or CD206 labeling. Scale bars, 50 μm. (E) Mean expression dot plot of Zinc transporter genes for each myeloid cluster. Size of circles indicate percentage of cells expressing the gene and increasing color gradient from white to red corresponds to increasing expression value. (F) Heatmap of mean AUCell enrichment of F4/80^hi/lo^ gene sets, corresponding to yolk sac (YS) versus hematopoetic stem-cell (HSC) lineage. Row enrichment value is scaled from 0 to 1 and presented as an increasing gradient from black, gray, yellow to orange which corresponds to increasing enrichment score. (G) Representative immunofluorescence microscopy images of cross sections of mouse prostate labeled for F4/80 (green), MHCII (red), CD11b (blue), CD31 (yellow) and phalloidin. Scale bars, 120 μm. (H) Cell counts per gram of prostate for rat IgG2a isotype or anti-Csf1r antibody (Ab) treated male mice. N = 5 per group. ^∗∗∗∗^p < 0.0001; n.s denotes not significant (p > 0.05) (Two-way ANOVA with Tukey’s multiple correction). (I) Zinc concentration of anterior prostate lobe, liver lobe, and kidney from male mice treated with either rat IgG2a isotype control or anti-Csf1r Ab. N = 6 per group. (shown is representative quantification from one of two independent experiments). ^∗^p < 0.05; n.s. not significant (Mann-Whitney test). See also Figures S4–S5.

We next considered MNPs in the human prostate in isolation and integrated scRNaseq data from MNPs in a published normal prostate dataset (Henry et al., 2018; Figure S4A). We found six distinct clusters of MNPs that exhibited transcriptional profiles consistent with their identity as monocytes (Mono), conventional DCs (cDCs), proliferating macrophages (MAC-cycling), and macrophages (MAC1, MAC2 and MAC-MT) (Figures 3A, S4B, and S4C). Two major subsets of cDCs are recognized, cDC1 and cDC2. cDC1 express XCR1 and cross-present antigens to CD8 T cells, while cDC2 express CD1c and activate CD4 T cells (Guilliams et al., 2014). In our dataset, we did not find two distinct DC clusters, but both *CD1C* positive and negative cells were evident within the DC cluster (Figure S4B). Two of the three macrophage populations were transcriptionally similar (MAC1 and MAC2) (Figure 3B), while MAC-MT cells formed a completely distinct cluster (Figure 3A) and expressed high levels of the metallothionein family genes (Figure 3B), encoding cysteine-rich proteins that bind divalent heavy metal ions and are involved in the cellular transport, storage, and metabolism of metal ions (Miles et al., 2000). High expression of metallothioneins has been noted in prostate cancer (Wei et al., 2008, Wang et al., 2018), but the MAC-MT cluster contained cells from both our dataset and that generated from normal, young human prostate tissue (Henry et al., 2018; Figure S4C), confirming that these cells exist in healthy prostate tissue in homeostasis. We also validated the presence of MAC-MT in the three other published scRNaseq datasets of both normal prostate and prostate cancer (Figures 3C and S4D).

Spatially, MAC-MT cell signatures colocalized with those of LE-KLK3 and LE-KLK4 (Figure S4E), and we confirmed the presence of an MT1+ CD68+ macrophage population in human prostate using both RNA scope and immunofluorescence microscopy, which were localized adjacent to luminal regions (Figure 3D). Zinc accumulation is controlled by two families of zinc transporters, the SLC39 (Zrt- and Irt-like proteins [ZIP]) that increase intracellular zinc, and the SLC30 (ZnT) proteins that lower zinc cellular levels (Liuzzi and Cousins, 2004). Interestingly, among prostate MNPs, the MAC-MT subset expressed the highest level of the zinc transporter genes, *SLC39A8* (ZIP-8) and *SLC30A1* (ZNT-1) (Figure 3E). MAC-MT cells were unique to the prostate, with no transcriptionally similar cells identified in other human organs (Figure S4F). Together, these data suggest that MAC-MT represent a prostate-specific macrophage subset, adapted to residency within the high zinc environment (Costello and Franklin, 2016).

Tissue macrophages arise prenatally from yolk sac (YS) or fetal liver progenitors, but are variably replaced postnatally by monocyte precursors that adopt tissue-specific transcriptional profiles (Ginhoux and Guilliams, 2016, Mass et al., 2016). All three macrophage clusters in human prostate showed transcriptional similarity to YS-derived macrophages (Schulz et al., 2012), as did the proliferating macrophage cluster, but MAC2 also enriched for the monocyte-derived macrophage signature (Figure 3F). Therefore, MAC1 and MAC-MT may represent prenatally seeded macrophage subsets, while MAC2 may be monocyte-derived, subsequently taking up a tissue-macrophage transcriptional signature. In keeping with this, mouse prostate contained both F4/80^high^CD11b^lo^ and F4/80^lo^CD11b^high^ macrophage subsets, (Figure S5A) identified as YS and hematopoetic stem cell (HSC)-derived subsets, respectively, in murine fate-mapping studies (Schulz et al., 2012). An analysis of a murine prostate scRNaseq dataset (Karthaus et al., 2020) also confirmed the presence of macrophage clusters with enrichment for both YS- and monocyte-derived macrophage signatures (Figure S5B). Anatomically, F4/80^high^ macrophages were located among luminal epithelial cells in mouse prostate (Figure 3G). Expression of zinc transporters and metallothionein genes was also evident in mouse prostate macrophages, particularly F4/80^hi^ cells enriching for the YS-derived macrophage signature (Figures S5C and S5D).

We therefore hypothesized that these prostate macrophages may contribute to zinc homeostasis in the organ. To test this, we used an anti-Csf1r antibody which effectively depleted prostate macrophages, particularly the F4/80^hi^ subset (Figure 3G, 3H, S5E, and S5F). This led to a significant reduction in prostate zinc concentration (Figure 3I), showing that prostate macrophages play a role in maintaining tissue zinc levels. The tissue zinc concentration in other organs was unaffected by macrophage depletion (Figures 3I and S5F), indicating this is a prostate specific macrophage function.

### Lymphoid immune landscape of human prostate

**Figure 4:**
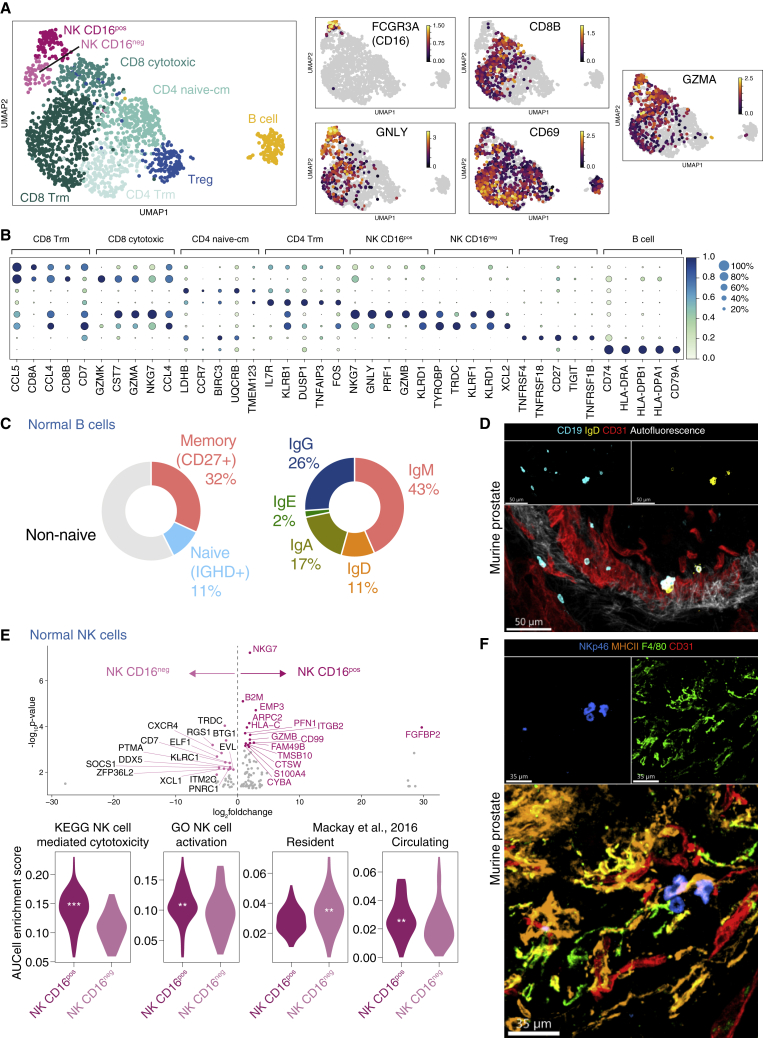
Lymphoid single-cell landscape of normal prostate and prostate cancer (A) UMAP of 1694 lymphoid cells from n = 7 patients. Expression of marker genes for NK cells (FCGR3A, GNLY), CD8 T cells (CD8B), tissue residency and activation (CD69) and cytolytic molecule (GZMA) are shown as a heatmap where gray indicates no expression and increasing expression is colored from purple, orange to yellow. (B) Dot plot of top five significant marker genes for each lymphoid clusters. Marker genes were identified using Wilcoxon rank sum test and p adj < 0.05 was considered statistically significant. Size of circles indicate percentage of cells expressing the gene and increasing color gradient from white to blue corresponds to increasing expression value. (C) Pie chart showing proportion of cells expressing markers for (left) memory (CD27+IGHD-), naive (IGHD+CD27-), non-naive (remainder) and (right) heavy gene constant gene expression. (D) Confocal imaging of CD19, IgG and CD31 in normal murine prostate section. Scale bars, 50 μm. (E) (Top) Volcano plot showing top 15 significant DEGs between NK CD16pos and NK CD16neg (normal only). (Bottom) Violin plots of gene set testing (AUCell) for NK cell gene sets (KEGG and GO) and lymphocyte tissue residency gene sets from (Mackay et al., 2016). Significance is denoted by ^∗∗^p < 0.01; ^∗∗∗^p < 0.001 (Mann-Whitney test). Position of asterisks indicate the group with higher expression. (F) Confocal imaging of NKp46, MHCII, F4/80 and CD31 in normal murine prostate section. Scale bars, 35 μm. See also Figure S6.

The lymphoid compartment of normal prostate included CD4 and CD8 T cells, two subsets of NK cells (CD16+ and CD16neg) and B cells (Figures 4A and 4B). Further analysis indicated the presence of naive, tissue-resident memory, and regulatory CD4 T cells, as well as cytotoxic and tissue-resident memory CD8 T-cell clusters (Figures 4A, 4B, and S6A) (Kumar et al., 2017) (Mackay et al., 2013) (Szabo et al., 2019). We categorized the B cells according to *CD27* (a marker of memory B cells) and *IGHD* (a marker of naive B cells) expression and observed that ∼30% and 10% of B cells were memory (*CD27+IGHD-)* and naive (*CD27-IGHD+*), respectively, with the remaining fraction mature non-naive B cells (Figures 4C and S6B). The majority of *IGHM*-expressing cells were CD27+ IgM memory cells, and there were also some class-switched B cells, with only a handful of *BLIMP1*-expressing plasma cells observed (Figure S6B). In the mouse prostate, extravascular naive and non-naive B cells were also evident adjacent to luminal epithelial cells (Figure 4D).

The identification of two subsets of NK cells in normal prostate is consistent with previous descriptions of NK cells in blood and other organs; the majority of peripheral blood NK cells are CD56^dim^CD16^**+**^, with a small subset of CD56^bright^CD16^neg^ NK cells, termed tissue-resident NK cells that are also present in spleen, uterus, and liver (Shi et al., 2011, Cuff et al., 2016). Functionally, these CD16^neg^ tissue-resident NK cells differ from conventional CD16^**+**^ NK cells, with reduced or altered cytotoxicity and prominent cytokine and chemokine production (Fauriat et al., 2010). In the prostate, CD16^**+**^ NK cells had a higher expression of genes associated with NK cell activation compared to CD16^neg^ NK cells, with significant enrichment of NK cell cytotoxicity and activation gene sets (Narni-Mancinelli et al., 2012; Figure 4E). In addition, CD16^neg^ NK cells showed enrichment for a universal lymphocyte tissue-residency signature (Mackay et al., 2016; Figure 4E), confirming that this subset represents tissue-resident NK cells and extending the list of tissues in which these cells have been identified in homeostasis (Dogra et al., 2020). The presence of CD16+ and CD16- subsets of NK cell was confirmed in an independent normal prostate single-cell dataset (Figure S6C), and NK cells were also evident in normal mouse prostate samples (Figure 4F).

### Immune perturbation in prostate cancer

**Figure 5:**
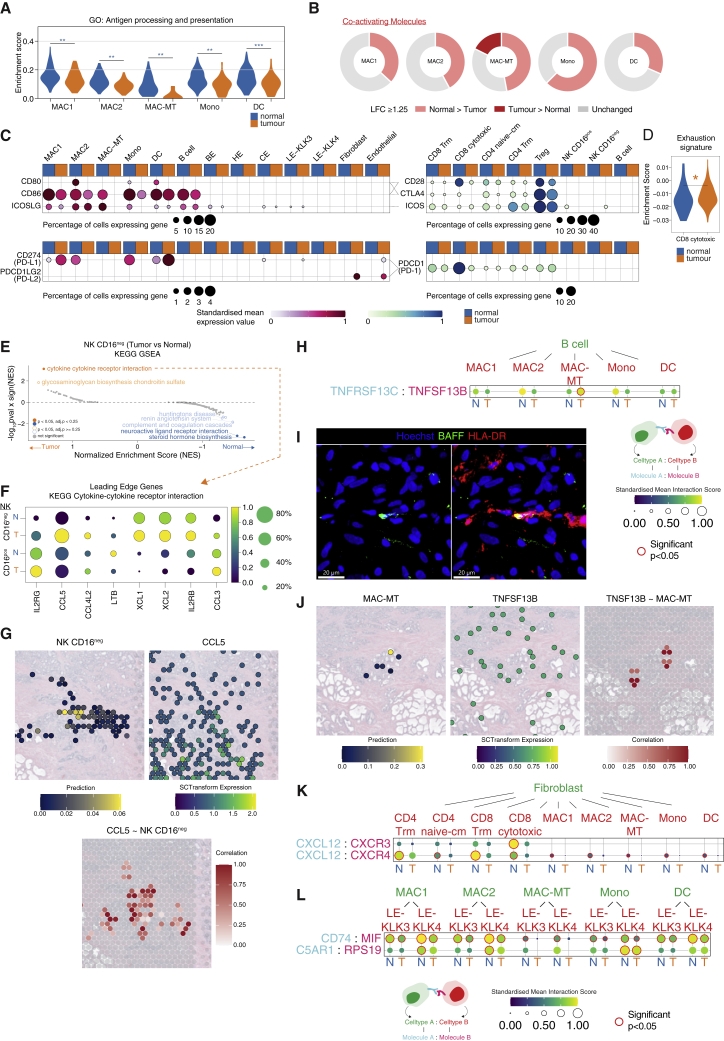
Perturbed immune cell transcriptomes and cellular interactions in prostate tumor (A) Violin plot of gene module score of GO term corresponding to antigen processing and presentation in tumor versus normal in myeloid cells. Kruskal-Wallis test was performed between normal and tumor for each cluster and p < 0.05 was considered statistically significant. (B) Pie chart of co-activating and co-inhibitory DEGs between normal (light colored sections) and tumor (dark colored sections) in myeloid clusters. Darker sections indicate genes that were that were upregulated in tumor versus normal and lighter section indicate upregulation is in normal versus tumor (> = 1.25 log_2_ fold change). Grey sections indicate < 1.25 log_2_ fold change. (C) Mean expression dot plot of costimulatory/coinhibitory molecules. Increasing expression corresponds with increasing gradient from white to red (MNP/B cell/epithelial/stromal) or white to blue (T/NK/B cell) corresponding to increasing expression value. Size of circles indicate the percentage of cells expression the gene. (D) Violin plot of gene set test (AUCell) results in CD8 cytotoxic T cell cluster for murine CD8 T cell exhaustion gene set curated from (Doering et al., 2012). Significance is denoted by ^∗^p < 0.05 (Mann-Whitney test). (E) GSEA of KEGG pathways for NK CD16^neg^ tumor versus normal. Statistically significant pathways are colored and labeled. (F) Mean expression dot plot of leading edge genes in cytokine-cytokine receptor interaction as in (E). Only genes that are expressed by at least 20% of cells are plotted. (G) (Top) Expression of CCL5 and prediction/label transfer score of NK CD16^neg^ cells in visium data of normal and tumor prostate sections. (Bottom) Spatial correlation of CCL5 with NK CD16^neg^ cells in prostate cancer visium data. Only positive correlations are plotted; increasing value of correlation is shown as a gradient from white to red. (H) CellPhoneDB receptor-ligand interaction analysis between B cell and myeloid clusters. (I) Representative immunofluorescence confocal microscopy of BAFF and HLA-DR in human prostate tumor section. Scale bars, 20 μm. (J–L) (J) Expression of TNFSF13B and prediction/label transfer score of MAC-MT cells, and correlation of TNFSF13B with MAC-MT cells in prostate cancer visium data. Only positive correlations are plotted; increasing value of correlation is shown as a gradient from white to red. CellPhoneDB receptor-ligand interaction analysis between (K) fibroblasts and T cell clusters, and fibroblast and myeloid clusters and (L) myeloid clusters with LE clusters split by group (N = normal; T = tumor). The order of the receptor-ligand interactions corresponds to the order of the cell-types i.e., cell type A expressing molecule A interacts with cell type B expressing molecule B. Size of circles and color gradient corresponds to the receptor-ligand interaction score, which purple, blue, green to yellow for increasing values. Significant interactions (p < 0.05) are highlighted in red. See also Figure S7.

We next sought to determine how prostate tissue immune cells were perturbed in cancer. Although there was no significant difference in immune cell number in prostate cancer samples (Figure 1E), several transcriptional differences in immune cells were evident; Antigen presentation and processing pathway genes were significantly reduced in prostate MNPs in cancer samples compared with normal prostate (Figure 5A), consistent with a widespread attenuation of CD4 T cell activation, a process critical for the generation of antitumor adaptive immune responses. In line with this, the expression of many co-activating receptors was higher in normal prostate MNPs compared with those in prostate cancer, the exception being MAC-MT, which showed higher expression of a subset of co-activating receptors in tumor samples, including CD40 (Figures 5B and S7A). There was increased expression of inhibitory *PDL1* in MAC1 and DCs in tumor (Figure 5C and S7A), little *PDL1* expression in non-immune –tumor cells, but some *PDL2* expression detectable in tumor fibroblasts and endothelial cells (Figures 5C, S7A, and S7B). In naive/T_cm_ CD4 T cells, there was reduced expression of *CD28*, *ICOS,* and *OX40* in tumor compared with normal, with little *PD1* expression and reduced *CTLA4* expression in tumor T cells (Figures 5C and S7A). Overall, in the lymphoid compartment, immune-related GO term genes were downregulated in tumor-associated lymphocyte subsets (Figure S7C), and there was significant enrichment for exhaustion signature genes in cytotoxic CD8 T cells (Figure 5D). Interestingly, in NK cells, we observed a significant enrichment of the *‘cytokine-cytokine receptor interaction’* gene set in the CD16^neg^ NK cell subset in tumor compared to normal (Figure 5E) and the leading-edge genes included several chemokine transcripts related to DC recruitment, including *CCL5*, *XCL1,* and *XCL2* (Figure 5F). Spatial transcriptomic analysis of prostate cancer confirmed colocalization of the CD16^neg^ NK cell signature and CCL5 transcripts (Figure 5G). This suggests that the CD16^neg^ resident-like NK cells in the prostate may promote the recruitment of tumor antigen cross-presenting cDC1, with potential beneficial anti-tumor effects, as described in melanoma, breast, and colon cancer (Böttcher et al., 2018).

Analysis of predicted MAC-MT interactions with other immune cells based on receptor-ligand expression using CellPhoneDB (Vento-Tormo et al., 2018) demonstrated that MAC-MT within tumor samples had increased expression of the gene encoding BAFF (*TNFSF13B*) with the potential to support BAFF-R-expressing B cells (Figures 5H and S7D). BAFF protein expression was evident in prostate adenocarcinoma samples (Figure S7E) and BAFF staining colocalized with MHCII staining, marking MNPs (Figure 5I-J). In contrast, analysis of immune-fibroblast interactions highlighted *CXCL12* expression by fibroblasts, with the potential to recruit *CXCR3/4*-expressing CD8 T cells and MNPs that was reduced in tumor (Figure 5K). LE-KLK3 and LE-KLK4 both expressed macrophage inhibitory factor (*MIF*), a macrophage survival, activation and recruiting factor (Gregory et al., 2006), with its receptor *CD74* expressed by all prostate MNPs, and this interaction was attenuated in cancer samples (Figure 5L). LE-KLK4 also expressed *RPS19*, with monocyte-recruiting activity (Yamamoto, 2007), and this too was reduced in tumor samples (Figures 5L and S7F).

In summary, these data indicate widespread immune transcriptional perturbation in prostate cancer, with reduced antigen presentation gene expression in MNP subsets, increased expression of exhaustion-associated genes in CD8 T cells, and reduced expression of immune-recruiting and activating chemokines and cytokines by fibroblast and epithelial cells in prostate cancer. However, in contrast, CD16^neg^ NK cells in tumor had increased expression of cDC1-recruiting chemokines and MAC-MT higher expression of the B cell survival factor BAFF, both with potential beneficial antitumor effects.

### MAC-MT in tumor associated with improved outcomes

**Figure 6:**
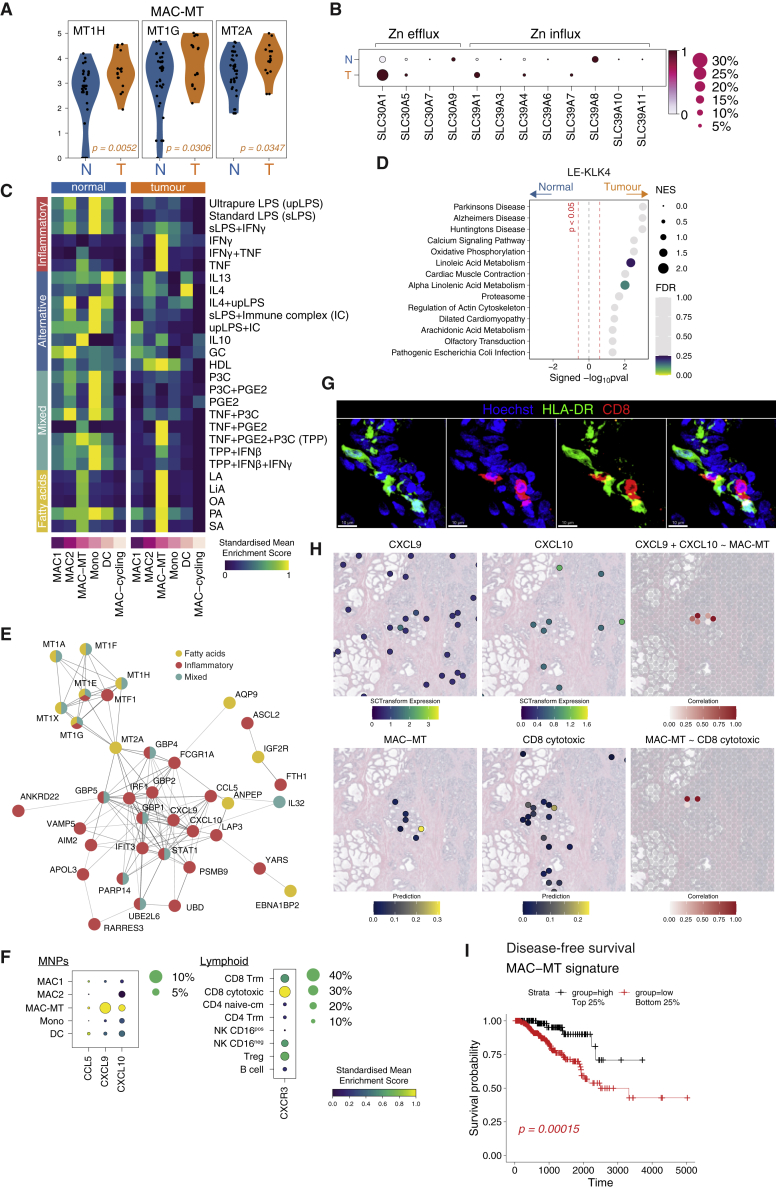
MT1-expressing macrophages in tumor have increased metallothionein and pro-inflammatory gene expression and are associated with improved tumor event-free survival (A) Violin plots show genes than achieved a p adj < 0.05 after statistical analyses with Wilcoxon Rank Sum Tests. Color of adjusted p value indicates the group where expression is higher (orange = tumor). (B) Mean expression dot plot of metal ion transport genes in MAC-MT cluster separated by normal (N) or tumor (T) in rows. Size of circle indicates the percentage of cells expressing the genes and color indicates which group (normal or tumor) expresses higher (dark red) levels of the genes. (C) Heatmap of mean AUCell enrichment of 27 macrophage-stimulation signatures split by normal or tumor. Row expression value is scaled from 0 to 1 and presented as a gradient from purple, blue, green to yellow. (D) GSEA of KEGG pathways in tumor versus normal for LE-KLK4. Pathways were considered statistically significant if p value < 0.05 (marked by vertical dashed red line). Size of circles indicate normalized enrichment score (NES) and colors indicate if pathways achieved FDR < 0.25 starting from purple, blue, green to yellow as significance values decreases. (E) String-DB analysis of leading edge genes from selected pathways enriched in tumor MAC-MT. (F) Mean expression dot plot of CCL5, CXCL9, CXCL10 in MNP clusters and CXCR3 in lymphoid clusters. Size of circle indicates the percentage of cells expressing the genes and increasing expression (scaled from 0 to 1) corresponds to increasing color gradient from purple, blue, green to yellow. (G) Representative immunofluorescence confocal microscopy of CD8 and HLA-DR in human prostate tumor section. Scale bars, 10 μm. (H) Expression of CXCL9 and CXCL10 and prediction/label transfer scores of MAC-MT and CD8 cytotoxic cells in visium data of tumor prostate sections. (Bottom) Spatial correlation of CXCL9 and CXCL10 with MAC-MT or MAC-MT with CD8 cytotoxic cells in prostate cancer visium data. Only positive correlations are plotted; increasing value of correlation is shown as a gradient from white to red. (I) Kaplan-Meier survival curve for TCGA-PRAD disease free index with deconvolved MAC-MT score. Samples were categorised into high (black, top 25%) and low (red, bottom 25%) of deconvolved score. Statistical analysis was performed with log rank test and p < 0.05 was considered statistically significant. See also Figures S8–S9 and Table S2.

To further probe the effect of the tumor microenvironment on MNPs, we compared GO term enrichment in normal and tumor MNPs. This analysis showed attenuated expression of all pathways enriched in homeostasis in every MNP subset except for MAC-MT (Figure S8A). Indeed, metallothionein genes were increased in tumor-associated MAC-MT compared with those in normal prostate (Figures 6A and S8B). *SLC30A1* expression was increased and *SLC39A8* decreased in MAC-MT cells in cancer compared to non-tumor samples (Figure 6B), the overall effect of which may be to increase zinc efflux, potentially counteracting the known decreased zinc concentration associated with prostate cancer.

Transcriptional alignment of healthy prostate MNPs with human macrophages activated with a variety of stimuli (Xue et al., 2014) demonstrated that MAC2 enriched for inflammatory LPS- and IFNγ-stimulated M1-like macrophage signatures, as well as IL4 and glucocorticoid-stimulated macrophage signatures (Figure 6C). MAC1 showed little enrichment for any signatures except for the glucocorticoid-stimulated macrophage signature, while MAC-MT were completely distinct, enriching for TNF-, IL10-, and fatty acid–stimulated macrophage signatures (Figure 6C). In contrast to normal prostate, MNPs in tumor tissue demonstrated a global reduction in most macrophage activation signatures, consistent with a broad immune-suppressive effect of the tumor environment (Figure 6C). However, the remarkable exception to this tumor-associated suppression was the MAC-MT subset, which showed a significant increase in the expression in IFNγ-, TNF-, and fatty acid–stimulated macrophage gene signatures (Figure 6C). We observed an increase in cholesterol homeostasis pathway genes (Hallmarks, Figure S8C) and linoleic acid (a fatty acid) metabolism pathway genes (KEGG, Figure 6D) in prostate cancer–associated LE-KLK4 cells, raising the possibility that the fatty acid response gene enrichment in MAC-MT cells may arise due to fatty acid generation by local LE-KLK4 cells. Of note, increased fatty acid production from *de novo* lipogenesis has been described in prostate cancer (Rossi et al., 2003) and inhibition of lipogenesis reduced cancer growth *in vitro* (Zadra et al., 2014).

STRING analysis of leading-edge genes (Figure S8D) in these macrophage activation signatures, showed that fatty acid stimulation genes upregulated in MAC-MT in tumor were dominated by metallothionine genes, while inflammatory stimulation genes included several chemokines (*CXCL9*, *CXCL10;* Figures 6E and 6F). CD8 cytotoxic T cells were the principle immune cell subset expressing *CXCR3*, the receptor for these chemokines (Figure 6F), suggesting that MAC-MT activation may promote CD8 T cell recruitment to tumors (Figure S8E). Consistent with this, some/many CD8 T cells were located adjacent to MNPs (Figure 6G) and spatial transcriptomic analysis of prostate cancer confirmed colocalization of the MAC-MT signature with *CXCL9/10* and with CD8 T cells (Figure 6H).

Given the pro-inflammatory transcriptional profile of MAC-MT cells, we hypothesized that they may have an antitumor effect, promoting anti-cancer immune responses and counteracting cancer-associated perturbations in zinc concentration. In keeping with this, cellular deconvolution (Figure S9A) of The Cancer Genome Atlas (TCGA) data (Figure S9B) indicated that prostate cancer biopsies with higher MAC-MT enrichment had a lower Gleason score (Figure S9C) and improved disease-free survival (Figure 6I; Table S2). No other individual immune cell subset signature had prognostic significance in this dataset, although the limited number of samples with a high CD16^neg^ NK cell signature precluded a robust analysis of the prognostic association of these cells (Figure S9C).

## Discussion

Our single-cell analysis of the human prostate delivered several remarkable findings; our sample processing protocol enriched for immune cells enabling us to deliver the most comprehensive overview of the immune landscape of normal human prostate to date. Our prostate immune cell atlas delineates a range of innate and adaptive immune cells, with several CD4 and CD8 T cell subsets, two subsets of NK cells, and a prostate-specific macrophage subset, which we designated MAC-MT. The latter subset was transcriptionally similar to YS-derived murine macrophages, consistent with the conclusion that they may be prenatally seeded, although transcriptional profile does not definitively prove ontogeny. They also expressed high levels of metallothionein and zinc transporter genes, suggesting that they could contribute to zinc homeostasis in the prostate. To test this hypothesis, we depleted prostate macrophages in mice, and confirmed a reduction in tissue zinc levels. In prostatic epithelial cells, the high zinc concentration acts to inhibit mitochondrial aconitase, truncating the Krebs cycle to generate citrate which is secreted into prostatic fluid maintaining spermatozoa (Costello and Franklin, 2016). The bioenergetic consequence of this is a reduction in ATP generation; therefore, aerobic glycolysis is increased in prostatic epithelium (Costello and Franklin, 2016). MAC-MT also enriched for glycolysis genes and may therefore directly contribute to prostate zinc and citrate via a similar mechanism, although we did not explore this in the current study. Of note, this homeostatic role of MAC-MT in maintaining prostate zinc adds to other examples of prenatally seeded tissue macrophages that contribute to organ physiology and function; for example, in the heart, a subset of macrophages act to buffer calcium ions within the conducting system (Hulsmans et al., 2017), and in the intestine, muscularis macrophages regulate the steady-state peristaltic activity of the colon (Muller et al., 2014).

In prostate cancer, the concentration of both citrate and zinc are markedly decreased (Franklin et al., 2005). We found that in prostate cancer, MAC-MT markedly increased the expression of the zinc efflux transporter *SLC30A1*, which may represent a mechanism to counteract the disrupted zinc transport present in tumor luminal epithelial cells. MAC-MT were also largely resistant to the immunosuppressive effect of the tumor environment. In fact, they become transcriptionally more inflammatory, expressing the B cell survival factor BAFF and lymphocyte-recruiting chemokines. Our analyses suggest that this response may be driven by the metabolic changes observed in KLK4 cells in tumor, namely the production of fatty acids. This increase in fatty acid metabolism has been previously described in prostate cancer (Rossi et al., 2003). We found that MAC-MT in tumor showed a transcriptional profile similar to that observed in macrophages stimulated with fatty acids. Remarkably, enrichment of the MAC-MT signature in prostate cancer biopsies was associated with improved disease-free survival, supporting the conclusion that their pro-inflammatory effects could be beneficial in the context of prostate cancer. The effects of MAC-MT enrichment are in contrast to previous studies investigating MNPs in prostate cancer which have associated the presence of myeloid-derived suppressor cells (Brusa et al., 2013) and CD163-positive M2 macrophages (Erlandsson et al., 2019) with worse survival, and emphasize the value of scRNA seq in delineating distinct cell subsets with important transcriptional and functional differences (Montoro et al., 2018). Our data have translational relevance as the antitumor effects of MAC-MT could potentially be harnessed as an immunotherapeutic strategy in advanced progressive prostate cancer.

We also identified a tissue-resident CD16^neg^ NK cell subset in normal prostate. These cells showed increased expression of *CCL5*, *XCL1*, and *XCL2* in prostate cancer, with the potential to recruit cDC1. This is reminiscent of a recent study in mice showing that NK cells in implanted melanomas, breast, and colon cancers played a critical role in recruiting cDC1 via the production of CCL5 and XCL1, with important antitumor effects (Böttcher et al., 2018). cDC1 express XCR1 (Dorner et al., 2009), as well as CCR1 and CCR5, both of which bind CCL5 (McColl, 2002) and have antitumor functions; they can attract and activate tumor-specific CD8 T cells (Spranger et al., 2017) (Broz et al., 2014), and internalize and transport tumor antigens to lymph nodes, where they may cross-prime CD8 T cells (Roberts et al., 2016). The potential importance of NK cells in antitumor responses in prostate cancer has been suggested by previous studies associating lower numbers of peripheral blood NK cells, particularly CD16^neg^ NK cells (Koo et al., 2013), or reduced activation capacity of circulating NK cells, with a higher risk of prostate cancer on biopsy (Vidal et al., 2019). NK cells within prostate cancer tissue have not been well studied, but enumeration via immunohistochemical staining indicated an association with a lower risk of disease progression (Gannon et al., 2009), and there is one previous flow cytometric assessment of prostate tumor NK cells, demonstrating an enrichment of the CD56^bright^ subset, relative to blood (Pasero et al., 2016). Our study confirms the presence of a tissue-resident CD56^bright^ CD16^neg^ NK cell subset in the prostate in health and in cancer, and provides a transcriptional assessment of these cells, revealing a potentially important antitumor function in cDC1 recruitment.

To date, metastatic prostate cancer has shown variable responsiveness to checkpoint blockade (Kwon et al., 2014, Beer et al., 2017). Evaluation of PD-L1 expression in prostate cancer previously identified its presence in less than 10% of primary cancer and up to a third of metastatic cancers (Haffner et al., 2018). Our analysis enabled simultaneous assessment of all PD1/2 ligands and receptors across cell types. *PDL1* was expression was upregulated in tumor MAC2 and DCs, and we identified *de novo PDL2* expression in tumor samples, suggesting that this may be a more relevant therapeutic target in prostate cancer than PDL1. This is consistent with a recent analysis of bulk RNA seq data from prostate cancer biopsies that found an increase in *PDL2* transcripts compared with normal tissue, and that higher *PDL2* expression was associated with worse outcomes (Zhao et al., 2019). Our analysis enabled the specific identification of tumor fibroblasts and endothelial cells as key expressors of this immunoinhibitory molecule.

Aside from tissue-resident immune cells, we also identified a subset of luminal epithelial cells that lack expression of AR that may interact metabolically with the prostate-specific MAC-MT. Our dataset delineates several cell-specific markers that should be investigated for their utility as biomarkers for the early identification of this cell subset to assess their contributions to development of castration-resistant disease.

In summary, we define the immune cell landscape in normal human prostate and describe its perturbation in cancer. Our study revealed the presence of a prostate-specific macrophage subset, marked by high expression of metallothionein genes that, in contrast to other MNP subsets, increases inflammatory gene expression in cancer with potential beneficial prognostic effects.

### Limitations of the study

While our study used a tissue processing strategy that enriched for immune cells on n = 10 paired normal and prostate cancer samples and successfully created a single-cell prostate immune atlas, there are several limitations; the patients recruited to our study had predominantly moderate prostate cancer and did not receive androgen deprivation therapy, in contrast to the other prostate cancer single-cell datasets which primarily consist of samples from more advanced disease. Furthermore, we discovered a metallothionein- expressing, zinc-regulating macrophage (MAC-MT) population, the MAC-MT gene signature was associated with improved outcomes, and experimental depletion in mice showed that prostate macrophages contribute to zinc homeostasis in normal prostate. However, further work is needed to investigate the role of these macrophages in the context of prostate cancer.

## STAR★Methods

### Key resources table

**Table:** 

Reagent or resource	Source	Identifier
**Antibodies**		

anti-human CD14 (FITC)	Invitrogen	Cat#11-0419-42; clone 61D3; RRID: AB_10597597
anti-human CD45 (APC-eFluor780)	eBioscience	Cat#47-0459-42; clone HI30; RRID: AB_1944368
anti-human CD19 (eFluor450)	eBioscience	Cat#48-0199-42; clone HIB19; RRID: AB_1272053
anti-human CD3 (BV785)	BioLegend	Cat#317330; clone OKT3; RRID: AB_2563507
anti-human CD8 (PE)	BioLegend	Cat#300908; clone HIT8a; RRID: AB_314112
anti-human CD16 (PE-Cyanine7)	Invitrogen	Cat#25-0168-42; clone CB16; RRID: AB_10714839
anti-mouse Ly-6G/Ly-6C (Gr1) (FITC)	BioLegend	Cat#108406; clone RB6-8C5; RRID: AB_313371
anti-mouse CD11b (PerCP-Cy5.5)	eBioscience	Cat#45-0112-82; clone M1/70; RRID: AB_953558
anti-mouse CD3e (APC)	eBioscience	Cat#17-0031-82; clone 145-2C11; RRID: AB_469315
anti-mouse CD19 (APC)	BioLegend	Cat#152410; clone 1D3; RRID: AB_2629839
anti-mouse CD45 (APC-eFluor780)	Invitrogen	Cat#47-0451-82; clone 30-F11; RRID: AB_1548781
anti-mouse I-A/I-E (Pacific Blue)	BioLegend	Cat#107620; clone M5/114.15.2; RRID: AB_493527
anti-mouse CD11c (PE)	BioLegend	Cat#117308; clone N418; RRID: AB_313777
anti-mouse F4/80 (PE-Cyanine7)	Invitrogen	Cat#25-4801-82; clone BM8; AB_469653
anti-mouse CD4 (FITC)	eBioscience	Cat#11-0041-82; clone GK1.5; RRID: AB_464892
anti-mouse NK1.1 (Pacific Blue)	BioLegend	Cat#108722; clone PK136; RRID: AB_2132712
anti-mouse CD8a (BV785)	BioLegend	Cat#100750; clone 53-6.7; RRID: AB_2562610
anti-mouse NKp46 (PE/Dazzle)	BioLegend	Cat#137630; clone 29A1.4; RRID: AB_2616666
anti-mouse CD3 (PE-Cyanine7)	BD PharMingen	Cat#560591; clone 17A2; RRID: AB_1727462
anti-mouse Ly-6G/Ly-6C (Gr-1) (APC)	BioLegend	Cat#108412; clone RB6-8C5; RRID: AB_313377
anti-mouse CSF1R	BioXCell	Cat#BE0213; clone AFS98; RRID: AB_2687699
Rat IgG2a isotype control	BioXCell	Cat#BE0089; clone 2A3; RRID: AB_1107769
anti-human HLA-DR (AF647)	Abcam	Cat#ab20181; clone TAL 1B5; RRID: AB_445401
anti-human CD206 (PE/Dazzle)	BioLegend	Cat#321130; clone 15-2; RRID: AB_2616867
anti-human MT1	Abcam	Cat#ab12228; clone UC1MT; RRID: AB_298949
anti-human CD3 (AF488)	BioLegend	Cat#300415; clone UCHT1; RRID: AB_389310
anti-human BAFF (polyclonal rabbit)	Bioss	Cat#bs-2431R; RRID: AB_10855666
Mouse IgG1 kappa monoclonal isotype control	Abcam	Cat#ab170190; clone 15-6E10A7; RRID: AB_2736870
Goat anti-mouse IgG secondary (FITC, polyclonal)	Invitrogen	Cat#31569; RRID: AB_228306
anti-mouse CD31 (AF594)	BioLegend	Cat#102520; clone MEC13.3; RRID: AB_2563319
anti-mouse F4/80 (AF647)	Abcam	Cat#ab204467; clone F4/80; RRID: AB_2810932
anti-mouse CD19 (AF594)	BioLegend	Cat#115552; clone 6D5; RRID: AB_2563459
anti-mouse IgD (AF488)	BioLegend	Cat#405718; clone 11-26c.2a; RRID: AB_10730619
anti-mouse NKp46 (PE)	eBioscience	Cat#12-3351-82; clone 29A1.4; RRID: AB_1210743
anti-mouse CD8 (FITC)	eBioscience	Cat#11-0081-82; clone 53-6.7; RRID: AB_464915
anti-mouse CD3 (Pacific Blue)	BioLegend	Cat#100214; clone 17A2; RRID: AB_493645
Flash Phalloidin 488	BioLegend	Cat#424201
Hoechst 33258	Biotium	Cat#40044
DAPI (in mounting medium)	Invitrogen	Cat#00-4959-52
LIVE/DEAD Aqua	Invitrogen	Cat#L34957

**Biological samples**		

Human normal and cancer prostate tissues	Cambridge University Hospitals NHS Foundation Trust	N/A

**Critical commercial assays**		

Zinc Assay Kit	Abcam	Cat#ab102507
Chromium Single Cell 3′ Library & Gel Bead Kit v2	10X Genomics	Cat#PN-120237
Chromium Single Cell A Chip Kit, 16 rxns	10X Genomics	Cat#PN-1000009
Chromium i7 Multiplex Kit, 96 rxns	10X Genomics	Cat#PN-120262
RNAscope® 2.5 LS Multiplex Reagent Kit	Advanced Cell Diagnostics	Cat#322800
RNAscope® LS 4-Plex Ancillary Kit Multiplex Reagent Kit	Advanced Cell Diagnostics	Cat#322830
RNAscope® 2.5 LS Probe- Hs-CD68-C2	Advanced Cell Diagnostics	Cat#560598-C2
RNAscope® 2.5 LS Probe- Hs-MT1-C3 (custom probe)	Advanced Cell Diagnostics	Cat#831088

**Deposited data**		

Matched normal and tumor prostate scRNaseq data	This paper, European Genome–Phenome Archive	EGAS00001005787, prostatecellatlas.org
Spatial Gene Expression Dataset by Space Ranger 1.3.0, Human Prostate Cancer, Adenocarcinoma with Invasive Carcinoma (FFPE)	10X Genomics	N/A
Prostate Cancer Spatial Transcriptomics data (L1.2) (Berglund et al., 2018)	European Genome–Phenome Archive	EGAS0000100300
Normal human prostate scRNaseq data (Henry et al., 2018)	GEO	GSE120716
Human prostate cancer scRNaseq data (Chen et al., 2021)	GEO	GSE141445
Human prostate cancer scRNaseq data (Crowley et al., 2020)	GEO	GSE150692
Human prostate cancer scRNaseq data (Karthaus et al., 2020)	https://singlecell.broadinstitute.org/	SCP864
Mouse prostate scRNaseq data (Karthaus et al., 2020)	GEO, https://singlecell.broadinstitute.org/	GSE146811, SCP859
Human prostate cancer Bulk RNaseq	TCGAbiolinks (R/Bioconductor)	TCGA-PRAD
Human spleen and lung scRNaseq data (Madissoon et al., 2019)	Human Cell Atlas Data Portal, https://www.tissuestabilitycellatlas.org/	N/A
Human kidney scRNaseq data (Stewart et al., 2019)	Human Cell Atlas Data Portal, https://www.kidneycellatlas.org/	N/A
Human liver scRNaseq data (MacParland et al., 2018)	GEO	GSE115469
Human heart scRNaseq data (Wang et al., 2020)	GEO	GSE109816

**Experimental models: Organisms/strains**		

Mouse: C57BL/6 (B6)	Jackson Laboratories	Stock No: 000664

**Software and algorithms**		

seurat	CRAN	V3.2.3
glmnet	CRAN	V4.1-2
survival	CRAN	V2.41-3
survminer	CRAN	V0.4.6
pheatmap	CRAN	V1.0.12
AUCell	Bioconductor	V1.14.0
fgsea	Bioconductor	V1.18.0
clusterProfiler	Bioconductor	V4.0.5
biomaRt	Bioconductor	V2.48.3
TCGAbiolinks	Bioconductor	V2.15.3
String-DB	https://string-db.org	V11
scanpy	https://github.com/theislab/scanpy	V1.4.5.post2 and V.1.7.2
soupx	https://github.com/constantamateur/soupx	V1.2.1
scrublet	https://github.com/swolock/scrublet	V0.2.1
umap	https://github.com/lmcinnes/umap	V3.10.0
gseapy	https://github.com/zqfang/gseapy	V0.10.5
stLearn	https://github.com/BiomedicalMachineLearning/stLearn	V0.3.2
CellPhoneDB	https://github.com/Teichlab/cellphonedb	V2.0.5
MuSiC	https://github.com/xuranw/MuSiC	V0.1.1
Cellranger	10X Genomics	V2.1.0
CASAVA	Illumina	V1.8.2
FlowJo	BD	V10

### Resource availability

#### Lead contact

Further information and requests for resources and reagents should be directed to and will be fulfilled by the Lead Contact, Menna R. Clatworthy (mrc38@cam.ac.uk).

#### Materials availability

This study did not generate new unique reagents.

### Experimental model and subject details

#### Participants

Fifteen men (50 – 74 years old) undergoing image guided prostate biopsies for suspicion of prostate cancer were enrolled in the DIAMOND study (NHS National Research Ethics Service reference 03/018) (CI:Gnanapragasam). All participants had previously undergone multi-parametric magnetic resonance imaging (mpMRI) of the prostate on a 3T magnet (Discovery MR750, GE Healthcare), using a 32-channel phased array coil. T2-weighted, contrast-enhanced, and diffusion-weighted imaging was acquired using the LIKERT scoring system according to Prostate Imaging–Reporting and Data System (PI-RADS) guidelines (Barrett et al., 2019). Only men with a positive MRI were approached and recruited for this study, defined as a PI-RADS score 3 or greater.

Each participant underwent transperineal biopsy under general anesthesia using the Biopsee fusion platform (Medcom, Darmstadt, Germany) according to the Ginsburg protocol, with a variable number of biopsies cores taken in order to obtain an appropriate tissue diagnosis for that individual (Kuru et al., 2013). All targets were defined by radiologists pre-procedure using T2-weighted imaging as the primary source images, using Biopsee fusion software. Patient/sample characteristics are summarized in Table S1. Samples from 5 men were used for sample preparation optimization and remaining 10 were used for sequencing experiments.

#### Mice

All murine research was conducted under the Animals (Scientific Procedures) Act 1986 Amendment Regulations 2012 following ethical review by the University of Cambridge Animal Welfare and Ethical Review Body (AWERB). Mice were housed at Cambridge Biomedical Services under specific-pathogen-free conditions. Wild-type C57BL/6 male mice aged 8 – 12 weeks were obtained from Jackson Laboratories (Margate, UK),

### Method details

#### Sample collection

Each participant underwent prostate biopsy with a variable number of biopsies (20-30) cores taken in order to obtain an appropriate tissue diagnosis. Samples were taken from systematic and targeted biopsies as standard of care. Men were consented to have additional cores taken from the “Target” (area where cancer was suspected on the MRI) and from an “off-target” area to provide a normal prostate tissue comparator. The number of biopsies taken from both areas ranged from 1 to 6 additional cores. Biopsies were placed into phosphate buffered saline and placed on ice immediately.

#### Tissue disaggregation of human tissue

Prostate tissue was received in ice cold PBS, minced into approximately 5 mm^3^ pieces and digested for 20 min at 37°C with agitation in a digestion solution containing 32.5 μg/mL Liberase TM and 50 μg/mL DNase in RPMI. Following incubation samples were passed through a 100 μm cell strainer using a 1 mL syringe plunger and washed by centrifugation with PBS. Live cells were enriched using a Dead Cell Removal kit (Miltenyi Biotec) as per manufactures instructions. This was followed by a 44% Percoll density-gradient for 30 min at room temperature. Enriched live cells were washed and counted using a haemocytometer with trypan blue. Cells were then blocked with human FcR block (Miltenyi Biotech) prior to surface staining for flow cytometry. Cell counts per gram were calculated with the addition of 123count eBeads.

#### Single-cell sequencing

10X Chromium Chip Single-cell library generation and preparation were performed on the single-cell suspension according to 10X Chromium 3′ solution (V2 kit) as per manufacturer’s instructions with an aim to capture 5000-10000 cells/channel. Sequencing was performed at the Cancer Research UK Cambridge Institute on the Illumina HiSeq4000 platform.

Following sequencing BCL files were demutiplexed to Fastq files using CASAVA. Subsequently splitting to single cells and mapping and quantification of genes was carried out using Cellranger software package (10X genomics). This generated count tables of unique molecular identifiers (UMI) for each gene per droplet.

#### Tissue disaggregation of murine tissue

The left and right anterior prostate lobes were harvested from mice and minced into approximately 15 mm^3^ pieces. Samples were digested for 20 min at room temperature in a digestion solution containing 0.1 M HEPES, 32.5 μg/mL Liberase TM and 50 μg/mL DNase in RPMI. Following incubation samples were passed through a 100 μm cell strainer using a 1 mL syringe plunger, washed by centrifugation with PBS and blocked with 50:50 mix of normal mouse and rat serum prior to staining. Cell counts per organ / gram of tissue were calculated with the addition of 123count eBeads (Invitrogen).

#### Flow cytometry

After blocking cells were incubated with live/dead cell staining (Live/Dead Aqua 405, Invitrogen) for 15 minutes on ice. Cell surface staining occurred on ice for 30 minutes. All samples were acquired on an LSR 4/5 laser Fortessa (BD) and data analyzed using FlowJo v10. *Human antibody:* anti-CD14 FITC (61D3, Invitrogen), anti-CD45 APC-eFluor780 (HI30, eBioscience), anti-CD19 eFluor450 (HIB19, eBioscience), anti-CD3 BV785 (OKT3, BioLegend), anti-CD8 PE (HIT8a, BioLegend), anti-CD16 PE-Cyanine7 (CB16, Invitrogen). *Murine antibody (myeloid panel):* anti-Gr1 FITC (RB6-8C5, BioLegend), anti-CD11b PerCP-Cy5.5 (M1/70, eBioscience), anti-CD3e APC (145-2C11, eBioscience), anti-CD19 APC (1D3, BioLegend), anti-CD45 APC-eFluor780 (30-F11, Invitrogen), anti-I-A/I-E Pacific Blue (M5/114.15.2, Biolegend), anti-CD11c PE (N418, BioLegend), anti-F4/80 PE-Cyanine7 (BM8, Invitrogen). *Murine antibody (lymphoid panel):* anti-CD4 FITC (GK1.5, eBioscience), anti-CD19 APC (1D3, BioLegend), anti-CD45 APC-eFluor780 (30-F11, Invitrogen), anti-NK1.1- Pacific Blue (PK136, BioLegend), anti-CD8a BV785 (53-6.7, BioLegend), anti-NKp46 PE/Dazzle (29A1.4, BioLegend), anti-CD3 PE-Cyanine7 (17A2, BD PharMingen).

#### Single-cell data analysis and preprocessing

The single-cell data (10X *cellranger* output) processed with EmptyDrops (Lun et al., 2019) and then corrected for ambient RNA expression using *SoupX* (v1.2.1) (Young and Behjati, 2020). Contamination fractions were estimated using the following genes: haemoglobin genes: *HBA1*, *HBA2* and *HBB*; immunoglobulin genes: *IGKC*, *IGLC1*, *IGLC2*, *IGLC3*, *IGLC4*, *IGLC5*, *IGLC6* and *IGLC7*; sperm genes: *STMN1*; prostate specific antigen gene: *KLK3*. *SoupX* was run with clustering information derived from a generic processing workflow in *Seurat* (Stuart et al., 2019). After *SoupX*, doublet detection was performed using *scrublet* (v0.2.1) (Wolock et al., 2019) with adaptations outlined in (Popescu et al., 2019) – Briefly, after *scrublet* was performed, the data was iteratively sub-clustered using standard *Seurat*-inspired *scanpy* (v.1.4.5.post2) workflow (Wolf et al., 2018, Stuart et al., 2019) and a median *scrublet* score for each sub-cluster was computed. Median absolute deviation (MAD) scores were computed from the cluster *scrublet* scores and a one tailed t test was performed with Benjamini-Hochberg (BH) correction (Benjamini and Hochberg, 1995) applied and cells with significantly outlying cluster *scrublet* scores (BH pval < 0.1) were flagged as potential doublets. The data was then processed using *scanpy* with standard quality control steps; cells were filtered if number of genes > 2500 or < 200. Percentage mitochondrial content cut-off was set at < 30%. Genes were retained if they are expressed by at least 3 cells. Genes counts for each cell were normalized to contain a total count equal to the median of total counts in cells before normalization. This led to a working dataset of 17,108 cells. We filtered out 1,616 cells that we could annotate as sperm cells from seminal fluid contamination from a single normal sample before resulting in the final set of 15,492 cells. Highly variable genes were selected based on the following parameters: minimum and maximum mean expression are > = 0.0125 and ≤ 3 respectively; minimum dispersion of genes = 0.5. The number of principal components used for neighborhood graph construction and dimensional reduction was set at 50. Batch correction was performed using *bbknn* with patients as the batch term with all other parameters as per default settings (Polański et al., 2020) (for all cells and for lymphoid analysis). Clustering was performed using Leiden algorithm (Traag et al., 2019) with resolution set at 1.0 (for all cells) or 0.5 (for myeloid and lymphoid). Henry et al. (Henry et al., 2018) dataset was identically processed for comparison except that cell type identities were used as published and sub-clustering of leukocytes was performed to extract MNPs after marker gene identification with Wilcoxon Rank Sum tests.

In all cases where Uniform Manifold Approximation and Projection (UMAP; v3.10.0) (McInnes et al., 2018) was used for dimensional reduction and visualization, the minimum distance was set at 0.3 and all other parameters as per default settings in *scanpy*.

For integration of MNPs, standard *SCTransform* workflow implemented in *Seurat* (v3.2.3) was used (Hafemeister and Satija, 2019). Calculation of PCA, UMAP and neighborhood graphs post integration was performed in *scanpy* using the *SCTransform* normalized data. Neighborhood graph graphs were constructed with 10 neighbors. For analysis of lymphoid cells, cells from three patients were excluded due to low numbers of cells (< 10 cells; D7 and D14) or low-quality information from cells (D6; insufficient cell-cell heterogeneity). Further sub-clustering was also performed on NK cells, CD8 T cells and non-CD8 T cells (annotated as CD4 T cells) separately to obtain the final lymphoid clusters via specifying the *restrict_to* option with resolution set at 0.3 in *scanpy*.

#### Differential gene testing

Differential gene testing was performed using the Wilcoxon test rank sum test implemented in *scanpy’s rank_genes_groups* module.

#### Cell type similarity assessment

We used a logistic regression approach to test for cell type similarity. This is done with L2-regularised logistic regression (ridge regression) multinomial models with the *glmnet* R package (Friedman et al., 2010) (i.e., alpha parameter = 0). Models were trained on normalized gene expression data with 10-fold cross-validation to obtain the appropriate *lamda* coefficient (*lambda.1se;* within 1 standard error from best model) for prediction. Gene expression values were standardized in both the training and test sets. The average of 50 iterations was used for the final score. To determine if predictions were significant, a median prediction probability score for each cluster was calculated and MAD-outliers were identified using a one-tailed t test. Cells were considered to be significantly similar if the BH p value was < 0.05 and the probability was > 50%. Re-embedding of each relevant dataset into UMAP or tSNE space were performed where possible with standard *scanpy* workflow and cluster identities were used as published. Label transfer for prostate cancer datasets (Chen et al., 2021, Crowley et al., 2020, Karthaus et al., 2020) was performed with default ingest protocol in implemented in *scanpy*. Pre-processing of these additional datasets were performed as close as possible to the dataset in this manuscript except for the exclusion of ambient RNA correction due to inavailability of raw data.

#### Gene set enrichment and pathway analyses

Gene module scores of gene sets used were obtained using *scanpy’s score_genes* module or AUCell (Aibar et al., 2017). Kruskal-Wallis test or Mann Whitney U tests were performed to test for significance of enrichment where appropriate using Prism software (v8). P value < 0.05 were considered as statistically significant. Typically, gene sets were retrieved and used as published in the original articles; in cases where murine gene sets were used, murine genes were converted to human orthologs using *biomaRt* (Durinck et al., 2009).

Pre-ranked gene set analysis (prGSEA) on hallmark genesets (Liberzon et al., 2015) and macrophage stimulation genesets (Murray et al., 2014) were performed with *gseapy* (https://github.com/zqfang/GSEApy/).

Gene ontology and KEGG pathway analyses were performed using *fgsea* (Korotkevich et al., 2019) or over-representation analysis implemented in *clusterProfiler* (Yu et al., 2012) R package. Genes were pre-ranked according to signed -log_10_Pvalues for all prGSEA procedures.

String-DB (v11) analysis was performed using the web browser tool (https://string-db.org/).

#### Spatial transcriptomics data analysis

We compared our scRNA-seq analysis with spatial gene expression data generated by spatial transcriptomics protocol (Ståhl et al., 2016) for prostate cancer tissues (Berglund et al., 2018). The tissues were from radical prostatectomy for a patient with adenocarcinoma. We used the tissue section L1.2, which was pathologically annotated as at cancer stage GLEASON score 3+3. For accurately mapping spot expression data to the tissue H&E image, we used spot count matrices with adjusted spot coordinates. The coordinate adjustment was based on the alignment of the H&E image with the corresponding spot-fluorescent image, where each spot was detected by Cy3 fluorescence signal. The alignment accounted for manufacturing variation that caused the differences between expected coordinates and the actual coordinates of spots printed onto the spatial gene-expression slide.

To classify cell-types in each spot, we used the anchor-based data integration method and calculated probabilistic transfer scores of discrete cell-type labels from cell types information in our reference scRNA-seq data to spot data (Stuart et al., 2019). For each spot, the class probability of the spot belonging to each of the 12 cell types was calculated. For estimating the abundance of two cell types in each spot as in the pie chart, we calculate the probability of the spot to be of the cell types of interest and scaled each value to 1. The script for anchor-based label transferring and for plotting cell type proportion to tissue is available at https://github.com/BiomedicalMachineLearning/stLearn and described in *stLearn* package (Pham et al., 2020).

For label transfer of the FFPE visium datasets available from 10X resource page, SCTransform was used to normalize both the reference (prostate single-cell dataset) and spatial data prior to integration as per instructions for Seurat v3.2.3. Expression values plotted are SCTransformed normalized values. For calculation of spatial correlation, k = 5 nearest neighborhoods were extracted from a kNN graph computed from the spatial location of each voxel. Peason’s correlation was then performed on each neighborhood using the gene expression value and cell type prediction value, followed by averaging across neighborhoods. Correlation values will not be returned if expression value was not detected in all neighborhoods or expression value was uniform across all voxels. For the CXCL9 + CXCL10 comparison, the value from *Seurat*’s *AddModuleScore* of the two genes was used for computing the correlation.

#### CellPhoneDB analysis

Normalized expression values from cell types found in this dataset were subjected to CellPhoneDB analysis (v2.0.0) (Efremova et al., 2020). The minimum threshold was set at 30% and results were considered statistically significant if p < 0.05.

#### Survival analysis

The Cancer Genome Atlas (TCGA) expression and clinical data for Prostate Adenocarcinoma (PRAD) were downloaded with TCGAbiolinks (v2.15.3) (Colaprico et al., 2016). Single-cell deconvolution was performed using Multi-subject Single Cell deconvolution R package (MuSiC, v0.1.1) (Wang et al., 2019) with raw counts as instructed in the package. Disease free survival indices were extracted from *days_to_new_tumor_event* contained in the TCGA clinical data; samples without *days_to_new_tumor_event* entries were removed. Events were considered if *days_to_new_tumor_event_dx* was annotated as ‘YES’ and all other samples were censored. Outcome for events were generally from biochemical evidence of disease but also included distant metastasis, locoregional recurrence, primary tumor and ‘*not available’*. All events were considered regardless of treatment received (radiological or pharmaceutical). Kaplan-Meier survival analyses were performed using the *survival* (v2.41-3) and *survminer* (v0.4.6) R packages where the deconvolved scores were categorised into a ‘high’ or ‘low’ group, which corresponds to top and bottom 25% MuSiC deconvolved scores. The results/info are tabulated and summarized in Table S2.

#### RNA *in situ* hybridization

Simultaneous detection of human CD68 and MT1 family genes were performed on FFPE sections using Advanced Cell Diagnostics (ACD) RNAscope® 2.5 LS Multiplex Reagent Kit (Cat No. 322800), RNAscope® LS 4-Plex Ancillary Kit Multiplex Reagent Kit (Cat No. 322830), RNAscope® 2.5 LS Probes (ACD, Hayward, CA, USA) at the histopathology/*in situ* hybridization core facility at Cancer Research UK – Cambridge Institute. Because it was not possible to design a specific RNAscope probe for Hs-MT1H due to high homology to other genes in the MT1 family, we used a probe that recognizes multiple MT1 genes. Briefly, sections were cut at 3 μm thick, baked for 1 h at 60°C before loading onto a Bond RX instrument (Leica Biosystems). Slides were deparaffinised and rehydrated on board prior to pre-treatments using Epitope Retrieval Solution 2 (Cat No. AR9640, Leica Biosystems) at 95°C for 15 min, and ACD Enzyme from the Multiplex Reagent kit at 40°C for 15 min. Probes were visualized using Opal fluorophores (Opal 570 and Opal 650 Akoya Biosciences Cat No. FP1488001KT and FP1496001KT respectively) diluted to 1:1000 using RNAscope LS Multiplex TSA Buffer. Probe hybridization, signal amplification and detection was performed on the Bond Rx according to the ACD protocol. Slides were then removed from the Bond Rx and mounted using Prolong Diamond (ThermoFisher Cat No P36965). The slides were imaged on the AxioScan (Zeiss) to create whole slide images. Images were captured at 40x magnification, with a resolution of 0.25 μm per pixel. ISH validation was carried out on one slide from the Cambridge 109 Prostate TMA (slide-1), consisting of paired sets of benign and tumor tissue cores that were collected under PrompT ethics (MREC/01/4/061).

#### In-vivo macrophage depletion

5-6 C57BL/6 male mice aged 16 weeks received 0.5 mg of depleting anti-CSF1R mAb (BioXCell, clone AFS98) or isotype control (BioXCell, Rat IgG2a) intraperitoneally on day −7, −4 and −2 prior to euthanasia per experiment (n = 2 independent replicates). Following terminal procedure, kidneys, liver and prostate from each mouse were harvested and divided for either flow cytometry, microscopy or zinc quantification. Tissues for flow cytometry was minced finely and digested in RPMI containing 0.1 mg/mL DNase I, 32.5 mg/mL Liberase TM and 10 mM HEPES for 25 min at room temperature. Organs were then mechanically dissociated through a 70 μm cell strainer, washed in PBS and red blood cell lysis performed. Single cell suspensions were blocked for 30 min with 50 μL normal mouse serum in PBS 2% FBS on ice then stained with live/dead fixable aqua (Invitrogen), anti-CD45 APC-eFluor780 (30-F11, eBioscience), anti-I-A/I-E Pacific Blue (M5/114.15.2, Biolegend), anti-CD11b PerCP-Cy5.5 (M1/70, Invitrogen), anti-F4/80 PE/Cyanine7 (BM8, Invitrogen), anti-CD3e APC (145-2C11, Invitrogen), anti-CD19 APC (1D3, eBioscience) and anti-Ly-6G/Ly-6C APC (Rb6-8C5, Biolegend).

#### Tissue zinc quantification

A single anterior prostate lobe, one kidney and one lobe of the liver per mouse was weighed and homogenized in 300 μL of T-PER lysis buffer using the Precellys homogenizer system. Samples were then centrifuged at 1500 x g for 10 min to remove contaminating material and supernatants used for zinc analysis. Zinc levels were analyzed using the Zinc Quantification Kit (ab102507, abcam) as per the manufacturer’s instructions. Zinc concentration expressed as nmol per gram of tissue.

#### Immunofluorescence microscopy

Samples were fixed in AntigenFix for 30 min at 4°C, rinsed in PBS for 5 min then transferred into 30% sucrose in PBS for 24 h and embedded in optimal cutting temperature compound for cutting. 30 μm sections were permeabilised and blocked in blocking buffer containing 0.1 M TRIS, 0.1% Triton, 1% normal mouse serum, 1% normal rat serum, 1% BSA for 1 h at room temperature. Staining was performed in blocking buffer for 2 h at room temperature prior to washing in PBS and mounting in Fluoromount-G or Fluoromount-G with DAPI. When required, a secondary staining was performed in blocking buffer for 2 h at room temperature prior to washing and mounting. Images were acquired using a TCS SP8 confocal microscope and raw images were processed using Imaris. Sections for the human sample were obtained from a 15 mm x 1 mm core needle biopsy as per sample collection described above. Sections for mouse sample were obtained from cross section of the prostate across the lateral/ventral and dorsal prostate region. Antibodies used include – mouse: MHC II-Pacific Blue (clone M5/114.15.2, 1/50 dilution, BioLegend), CD11b-PE (clone M1/70, 1/50 dilution, Invitrogen), CD31-AF594 (clone MEC13.3, 1/100 dilution, BioLegend), F4/80-AF647 (clone F4/80, 1/50 dilution, Abcam), CD19-AF594 (clone 6D5, 1/100 dilution, BioLegend), IgD-AF488 (clone 11-26c.2a, 1/50 dilution, Biolegend), NKp46-PE (clone 29A1.4, 1/50 dilution, eBioscience), CD8-FITC (clone 53-6.7, 1/50 dilution, eBioscience), CD3-Pacific Blue (clone 17A2, 1/50 dilution, BioLegend); human: HLA-DR-AF647 (clone TAL 1B5, 1/50 dilution, Abcam), CD206-PE Dazzle (clone 15-2, 1/50 dilution, BioLegend), MT1 (clone UC1MT, 1/50 dilution, Abcam), CD8-PE (clone HIT8a, dilution 1/100, BioLegend), CD3-AF488 (clone UCHT1, dilution 1/100, BioLegend), BAFF (polyclonal rabbit, dilution 1/50, Bioss), Mouse IgG1 kappa monoclonal isotype control (clone 15-6E10A7, 1/50 dilution, abcam), Goat anti-mouse IgG secondary-FITC (polyclonal, 1/200 dilution, Invitrogen). Dyes: Flash Phalloidin 488 (1/300 dilution, BioLegend), Hoechst 33258 (dilution 1/10 000, cat# 40044, Biotum), DAPI (in mounting medium, cat# 00-4959-52, Invitrogen).

#### Other data visualization

Results were generated using R packages or python modules and organized as figures using Adobe Illustrator. Combined scatter-,box-, violin-plots were generated in R using code based on (Allen et al., 2018) and violin plots and dot plots were generated using plotting modules implemented in *scanpy* or with R *ggplot2*-based functions. Heatmaps were generated using *pheatmap* (v1.0.12) R package or *matplotlib* (v3.0.3) modules in python. Bar plots were generated using Prism (v8).

### Quantification and statistical analysis

Statistical analysis was performed using GraphPad Prism software, R, or python and have been described in the relevant methods sections and figure legends accordingly. In general, unless otherwise specified, non-parametric tests were used and p value after false discovery correction procedures < 0.05 were considered statistically significant. Sample sizes for mice experiments can be found in figure legends.

### Additional resources

An interactive version and h5ad files of the single-cell RNaseq data is available at prostatecellatlas.org.

## Data Availability

• The raw single-cell RNA-sequencing data reported in this paper is deposited at the European Genome-Phenome Archive under the accession id EGA: EGAS00001005787 with restricted data access control and will be made available by the data access committee, including the lead contact, upon reasonable request. The count data and single-cell objects are available at www.prostatecellatlas.org. • All code used for the study are available at https://github.com/clatworthylab/prostateimmuneatlas. • Any additional information required to reanalyze the data reported in this paper is available from the lead contact upon request.

## Supplemental information

Document S1. Figures S1–S9

Table S1. Patient and sample information, related to Figure 1

Table S2. TCGA survival analysis information, related to Figure 6

Document S2. Article plus supplemental information
