# Supplementary material for: Resolving the immune landscape of human prostate at a single-cell level in health and cancer
Source: Cell Rep. 2021 Dec 21;37(12):110132. doi: 10.1016/j.celrep.2021.110132 (PMC8721283; doi:10.1016/j.celrep.2021.110132)
Supplement: Document S1. Figures S1–S9 [file mmc1.pdf]

Cell Reports, Volume *37*

Supplemental information

Resolving the immune landscape of human prostate at a single-cell level in health and cancer

Zewen Kelvin Tuong, Kevin W. Loudon, Brendan Berry, Nathan Richoz, Julia Jones, Xiao Tan, Quan Nguyen, Anne George, Satoshi Hori, Sarah Field, Andy G. Lynch, Katarzyna Kania, Paul Coupland, Anne Babbage, Richard Grenfell, Tristan Barrett, Anne Y. Warren, Vincent Gnanapragasam, Charlie Massie, and Menna R. Clatworthy

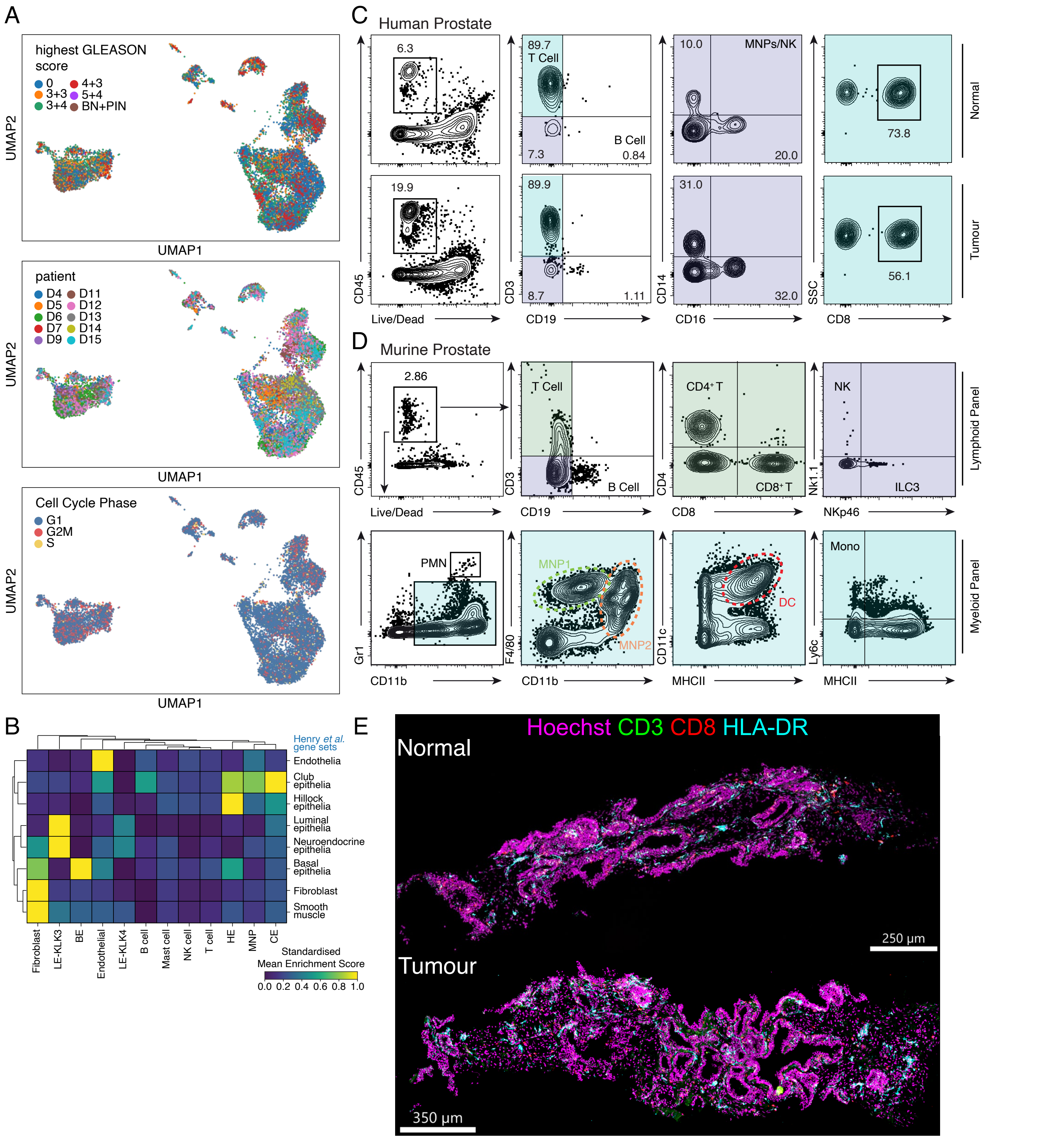

A
highest GLEASON score
0
4+3
3+3
5+4
3+4
BN+PIN
UMAP2
UMAP1
patient
D4
D11
D5
D12
D6
D13
D7
D14
D9
D15
Cell Cycle Phase
G1
G2M
S
B
Henry et al. gene sets
Endothelia
Club epithelia
Hillock epithelia
Luminal epithelia
Neuroendocrine epithelia
Basal epithelia
Fibroblast
Smooth muscle
Fibroblast
LE-KLK3
BE
Endothelial
LE-KLK4
B cell
Mast cell
NK cell
T cell
HE
MNP
CE
Standardised Mean Enrichment Score
0.0 0.2 0.4 0.6 0.8 1.0
C
Human Prostate
6.3
89.7
T Cell
10.0
MNPs/NK
B Cell
7.3
0.84
20.0
73.8
Normal
19.9
89.9
31.0
8.7
1.11
32.0
56.1
Tumour
CD45
Live/Dead
CD3
CD19
CD14
CD16
SSC
CD8
D
Murine Prostate
2.86
T Cell
CD4⁺ T
NK
B Cell
CD8⁺ T
ILC3
Lymphoid Panel
CD45
Live/Dead
CD3
CD19
CD4
CD8
Nk1.1
NKp46
PMN
MNP1
MNP2
DC
Mono
Myeloid Panel
Gr1
CD11b
F4/80
CD11b
CD11c
MHCII
Ly6c
MHCII
E
Hoechst CD3 CD8 HLA-DR
Normal
250 µm
Tumour
350 µm

Fig. S1

Fig. S1. Sample demographics, immune cell composition and histology of normal and tumor prostate, related to Fig. 1. (A) UMAP plot of prostate cells coloured by highest gleason score, patient (n=10), and assigned cell cycle phase (G1, G2M, S). BN+PIN denotes basal neoplasia+prostate intraepithelial neoplasia. (B) Heatmap of mean AUCell enrichment of marker genes from (Henry et al., 2018). Enrichment value is scaled row-wise from 0 to 1 and presented as an increasing gradient from purple, blue, green to yellow which corresponds to increasing enrichment score. (C) Gating strategy for immune subsets in normal and malignant prostate samples from human donors. Sequential gating was used to identify immune subsets and denoted by the same-coloured gate. Cell identity (gated on Live CD45+) – T cells (CD3+), CD8+ T (CD3+CD8+), CD4+ T (CD3+CD4+), B cells (CD19+), classical monocytes (CD14+CD16-) and non-classical monocytes (CD14-CD16+). (D) Gating strategy for immune subsets in murine prostate. Sequential gating was used to identify immune subsets and denoted by the same-coloured gate. Cell identity (gated on Live CD45+) – T cells (CD3+), CD8+ T (CD3+CD8+), CD4+ T (CD3+CD4+), B cells (CD19+), NK cells (CD3-NK1.1+), ILC3 (CD3-NKp46+), neutrophils (CD11bhiGr1+), MNP1 (F4/80hiCD11bint), MNP2 (F4/80intCD11bhi), dendritic cells (CD11c+MHCII+), monocytes (Ly6C+MHCII−) and macrophages (Ly6C−MHCII+). (E) Confocal imaging of CD3, CD8 and HLA-DR in normal and tumor human prostate Scale bars = 250 and 350 μm respectively.

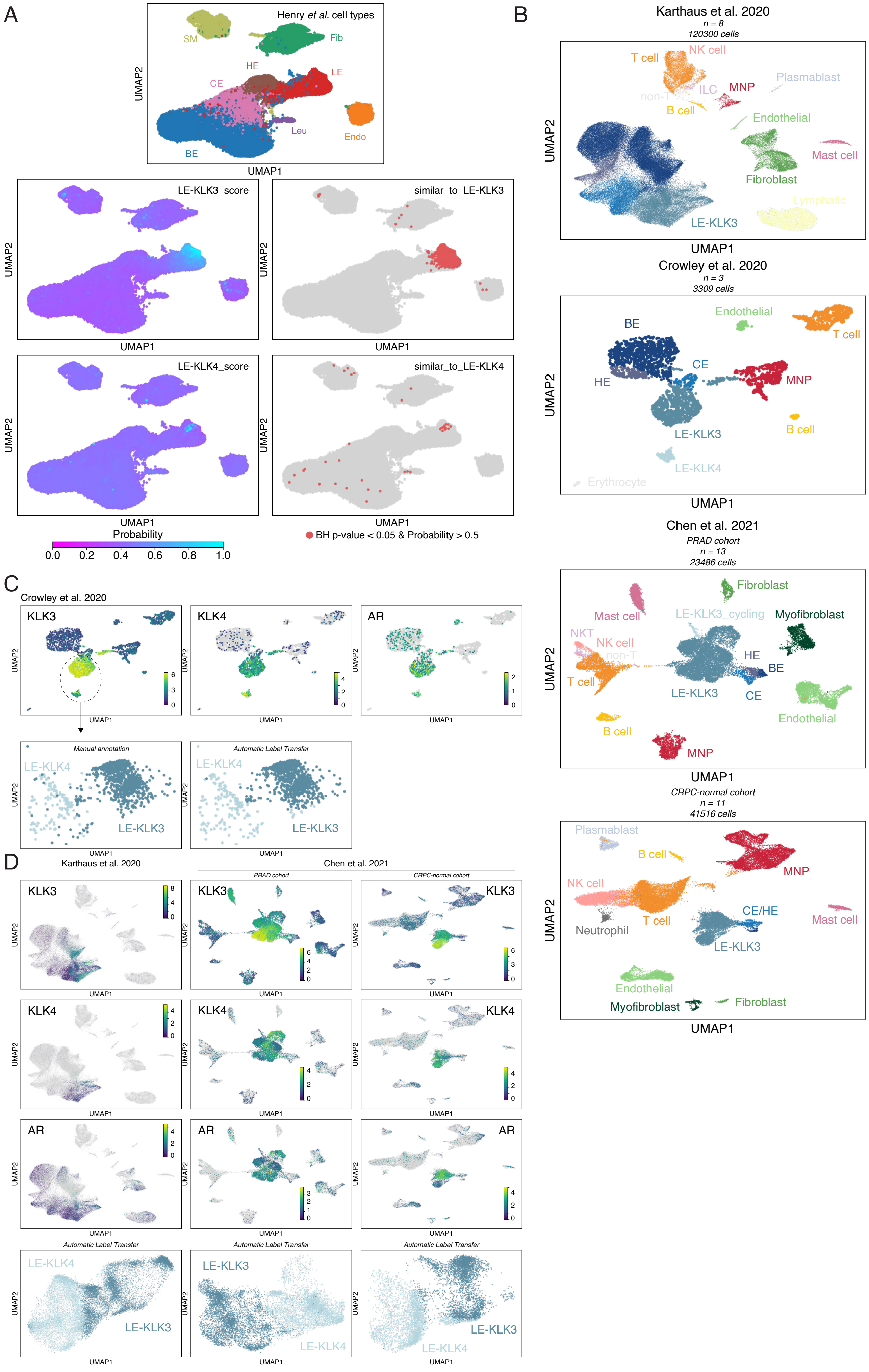

A
Henry et al. cell types
SM
Fib
HE
LE
CE
Leu
Endo
BE
UMAP1
UMAP2
LE-KLK3_score
similar_to_LE-KLK3
LE-KLK4_score
similar_to_LE-KLK4
Probability
0.0 0.2 0.4 0.6 0.8 1.0
BH p-value < 0.05 & Probability > 0.5
B
Karthaus et al. 2020
n = 8
120300 cells
T cell
NK cell
Plasmablast
ILC
MNP
non-T
B cell
Endothelial
Mast cell
Fibroblast
Lymphatic
LE-KLK3
Crowley et al. 2020
n = 3
3309 cells
Endothelial
BE
T cell
CE
HE
MNP
LE-KLK3
B cell
LE-KLK4
Erythrocyte
Chen et al. 2021
PRAD cohort
n = 13
23486 cells
Fibroblast
Mast cell
LE-KLK3_cycling
Myofibroblast
NKT
NK cell
non-T
HE
BE
T cell
LE-KLK3
CE
Endothelial
B cell
MNP
CRPC-normal cohort
n = 11
41516 cells
Plasmablast
B cell
MNP
NK cell
CE/HE
T cell
Mast cell
Neutrophil
LE-KLK3
Endothelial
Myofibroblast
Fibroblast
C
Crowley et al. 2020
KLK3
KLK4
AR
Manual annotation
Automatic Label Transfer
LE-KLK4
LE-KLK3
D
Karthaus et al. 2020
Chen et al. 2021
PRAD cohort
CRPC-normal cohort

Fig. S2

Fig. S2. Comparison with previous prostate single-cell data, related to Fig. 2. (A) UMAP plot of Henry *et al*., (Henry et al., 2018) cell types. Logistic regression results (probability) are coloured according to increasing gradient from purple to blue, corresponding to 0% to 100% similarity. Cells that achieved MAD-outlier BH p value < 0.05 and probability $> 50\%$ are highlighted in red. (B) UMAP embedding of prostate cancer single-cell data from (Karthaus et al., 2020, Chen et al., 2021, Crowley et al., 2020) with manual reannotation. (C-D) UMAP expression plot of KLK3, KLK4 and AR in data from (C) (Crowley et al., 2020) and (D) (Chen et al., 2021, Karthaus et al., 2020). Increasing colour gradient from blue to green to yellow corresponds to increasing expression value. Grey indicates no expression. Automatic label transfer result of luminal epithelial cells using *scanpy.tl.ingest* procedure is shown in bottom panels (manual annotation for (Crowley et al., 2020) is also shown in (C)).

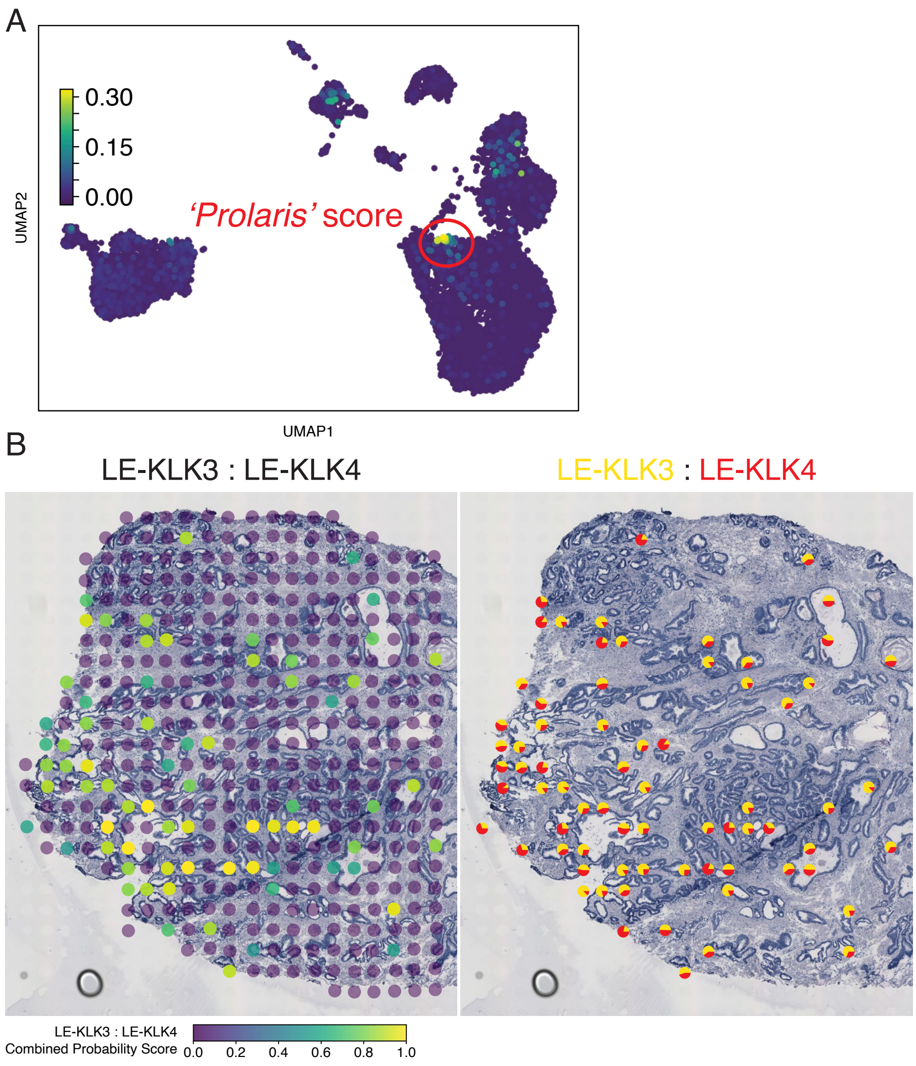
A
0.30
0.15
0.00
'Prolaris' score
UMAP2
UMAP1
B
LE-KLK3 : LE-KLK4
LE-KLK3 : LE-KLK4
LE-KLK3 : LE-KLK4
Combined Probability Score
0.0
0.2
0.4
0.6
0.8
1.0

Fig. S3

Fig. S3. Enrichment of prostate cancer signature and spatial correlation of luminal celltypes, related to Fig. 2. (A) UMAP enrichment score of 31 genes contained in 'Prolaris' prostate cancer aggressiveness/proliferation signature. (B) Prediction of cell subtypes in spatial transcriptomics data on prostate section (GLEASON score 3+3) (Berglund et al., 2018). (Left) Sum of probability scores in each spot contributed by LE-KLK3 and LE-KLK4 is shown as a heatmap. Increasing colour gradient from purple, blue, green to yellow corresponds to increasing (standardised) probability value. (Right) For estimating the abundance of two cell types in each spot as in the pie chart, we calculate the probability of the spot to be LE-KLK3 or LE-KLK4, setting a threshold for transfer score above 0.5 for each cell type. Contributions of LE-KLK3 (yellow) and LE-KLK4 (red) gene signatures in colocalised spots presented as a pie chart.

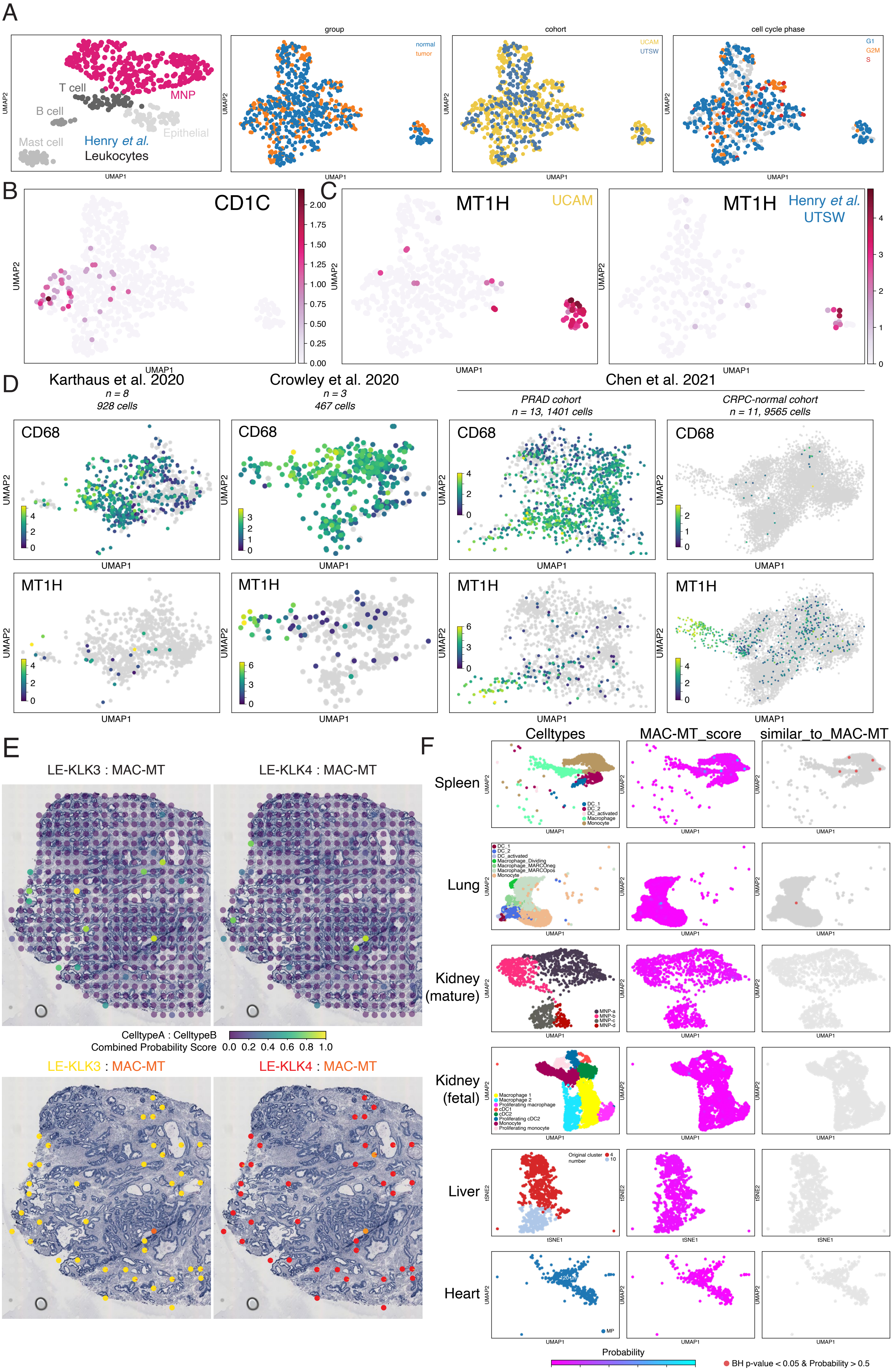

A
group
cohort
cell cycle phase
T cell
MNP
B cell
Epithelial
Mast cell
Henry et al.
Leukocytes
normal
tumor
UCAM
UTSW
G1
G2M
S
UMAP1
UMAP2
B
CD1C
C
MT1H
UCAM
MT1H
Henry et al.
UTSW
D
Karthaus et al. 2020
n = 8
928 cells
Crowley et al. 2020
n = 3
467 cells
Chen et al. 2021
PRAD cohort
n = 13, 1401 cells
CRPC-normal cohort
n = 11, 9565 cells
CD68
MT1H
E
LE-KLK3 : MAC-MT
LE-KLK4 : MAC-MT
CelltypeA : CelltypeB
Combined Probability Score
0.0 0.2 0.4 0.6 0.8 1.0
LE-KLK3 : MAC-MT
LE-KLK4 : MAC-MT
F
Celltypes
MAC-MT_score
similar_to_MAC-MT
Spleen
Lung
Kidney (mature)
Kidney (fetal)
Liver
Heart
Probability
0.0 0.2 0.4 0.6 0.8 1.0
BH p-value < 0.05 & Probability > 0.5

Fig. S4

Fig. S4. Integration of MNP data and comparisons to other single-cell data, related to Fig. 3. (A) (Left) UMAP plot of reclustered Henry *et al*., (Henry et al., 2018) immune cell types. (Right) UMAP plot of integrated myeloid data coloured according to group (normal vs tumour), study cohort and cell cycle phase (G1, G2/M or S; grey dots are cells from Henry *et al*.). (B) UMAP expression plot of CD1C expression in myeloid cells. Increasing colour gradient from white to red corresponds to increasing expression value. (C) UMAP expression plot of MT1H expression in myeloid cells split by study cohort. Increasing colour gradient from white to red corresponds to increasing expression value. (D) UMAP expression plot of CD68 and MT1H expression in myeloid cells from (Karthaus et al., 2020, Chen et al., 2021, Crowley et al., 2020). Increasing colour gradient from blue to green to yellow corresponds to increasing expression value. Grey indicates no expression. (E) Prediction of cell subtypes in spatial transcriptomics data on prostate section (GLEASON score 3+3) (Berglund et al., 2018). (Top) Sum of probability scores in each spot contributed by (left) LE-KLK3 and MAC-MT or (right) LE-KLK4 and MAC-MT are shown as a heatmap. Increasing colour gradient from purple, blue, green to yellow corresponds to increasing (standardised) probability value. (Bottom) For estimating the abundance of two cell types in each spot as in the pie chart, we calculate the probability of the spot to be LE-KLK3 (yellow), LE-KLK4 (red) or MAC-MT (orange), setting a threshold for transfer score above 0.5 for each cell type. (F) Logistic regression prediction of MAC-MT in human spleen and lung (Madissoon et al., 2019), kidney (Stewart et al., 2019), liver (MacParland et al., 2018) and heart (Wang et al., 2020) single-cell data sets. Only myeloid cells from the external data sets were considered for the analysis. Results (probability) are coloured according to increasing gradient from purple to blue, corresponding to 0% to 100% similarity. Cells that achieved MAD-outlier BH p value < 0.05 and probability $> 50\%$ are highlighted in red.

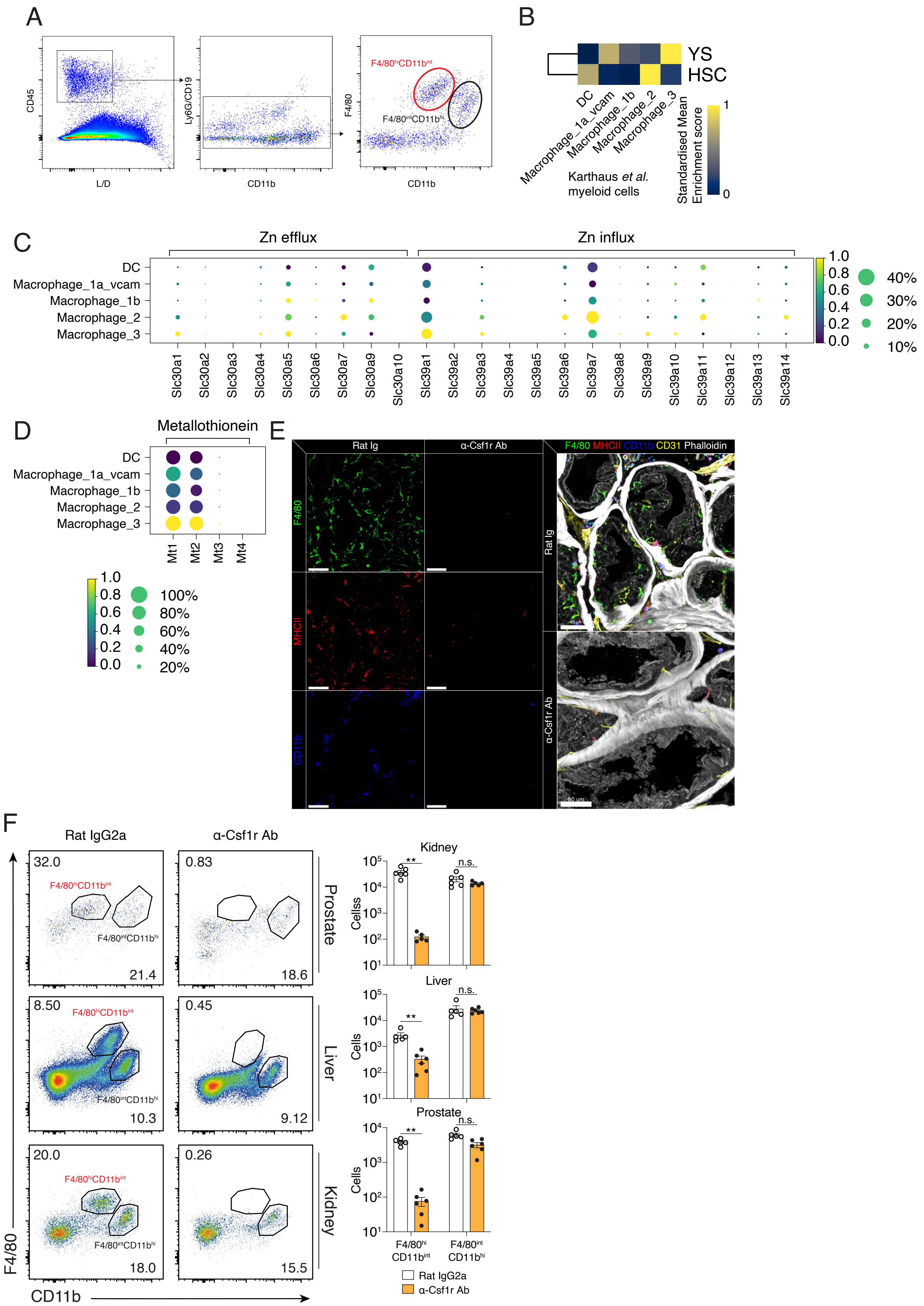
A
CD45
L/D
Ly6G/CD19
CD11b
F4/80
F4/80hiCD11bint
F4/80intCD11bhi
B
YS
HSC
DC
Macrophage_1a_vcam
Macrophage_1b
Macrophage_2
Macrophage_3
Karthaus et al.
myeloid cells
Standardised Mean
Enrichment score
C
Zn efflux
Zn influx
Slc30a1
Slc30a2
Slc30a3
Slc30a4
Slc30a5
Slc30a6
Slc30a7
Slc30a9
Slc30a10
Slc39a1
Slc39a2
Slc39a3
Slc39a4
Slc39a5
Slc39a6
Slc39a7
Slc39a8
Slc39a9
Slc39a10
Slc39a11
Slc39a12
Slc39a13
Slc39a14
40%
30%
20%
10%
D
Metallothionein
Mt1
Mt2
Mt3
Mt4
100%
80%
60%
40%
20%
E
Rat Ig
α-Csf1r Ab
F4/80 MHCII CD11b CD31 Phalloidin
F4/80
MHCII
CD11b
F
Rat IgG2a
α-Csf1r Ab
32.0
21.4
0.83
18.6
Prostate
8.50
10.3
0.45
9.12
Liver
20.0
18.0
0.26
15.5
Kidney
F4/80
CD11b
Kidney
Liver
Prostate
Cellss
Cells
**
n.s.
F4/80hi CD11bint
F4/80int CD11bhi
Rat IgG2a
α-Csf1r Ab

Fig. S5

Fig. S5. Examination of MNPs in murine prostate, related to Fig. 3. (A) Representative flow plots of CD45+ lineage-(CD19+, Ly6g+) murine prostate myeloid cells expressing CD11b and F4/80. (B) Heatmap of mean AUCell enrichment of F4/80$^{hi/lo}$ gene sets for myeloid cell types in (Karthaus et al., 2020), corresponding to yolk sac (YS) vs haematopoetic stem-cell (HSC) lineage. Row enrichment value is scaled from 0 to 1 and presented as an increasing gradient from blue, black to yellow which corresponds to increasing enrichment score. (C-D) Mean expression dot plot of (C) zinc transporters and (D) metallothionein genes in murine macrophage. Size of circle indicates the percentage of cells expressing the genes and increasing expression (scaled from 0 to 1) corresponds to increasing colour gradient from purple, blue, green to yellow. (E) Representative immunofluorescence microscopy images of cross sections of mouse prostate labelled for F4/80 (green), MHCII (red), CD11b (blue), CD31 (yellow) and phalloidin. Scale bars = 80 μm. (F) (left) Representative flow-cytometry plots of prostate, liver and kidney from male mice treated with rat IgG2a isotope or anti-Csf1r antibody. Percentage F4/80^{hi}CD11b^{int} and F4/80^{int}CD11b^{hi} are denoted by numerical value in upper left and lower right corner of plot respectively. (right) Quantification of absolute cell counts for prostate, liver and kidney by flow-cytometry for left panel. Each dot represents an individual animal. **$p<0.01$; n.s., not significant (Mann-Whitney test).

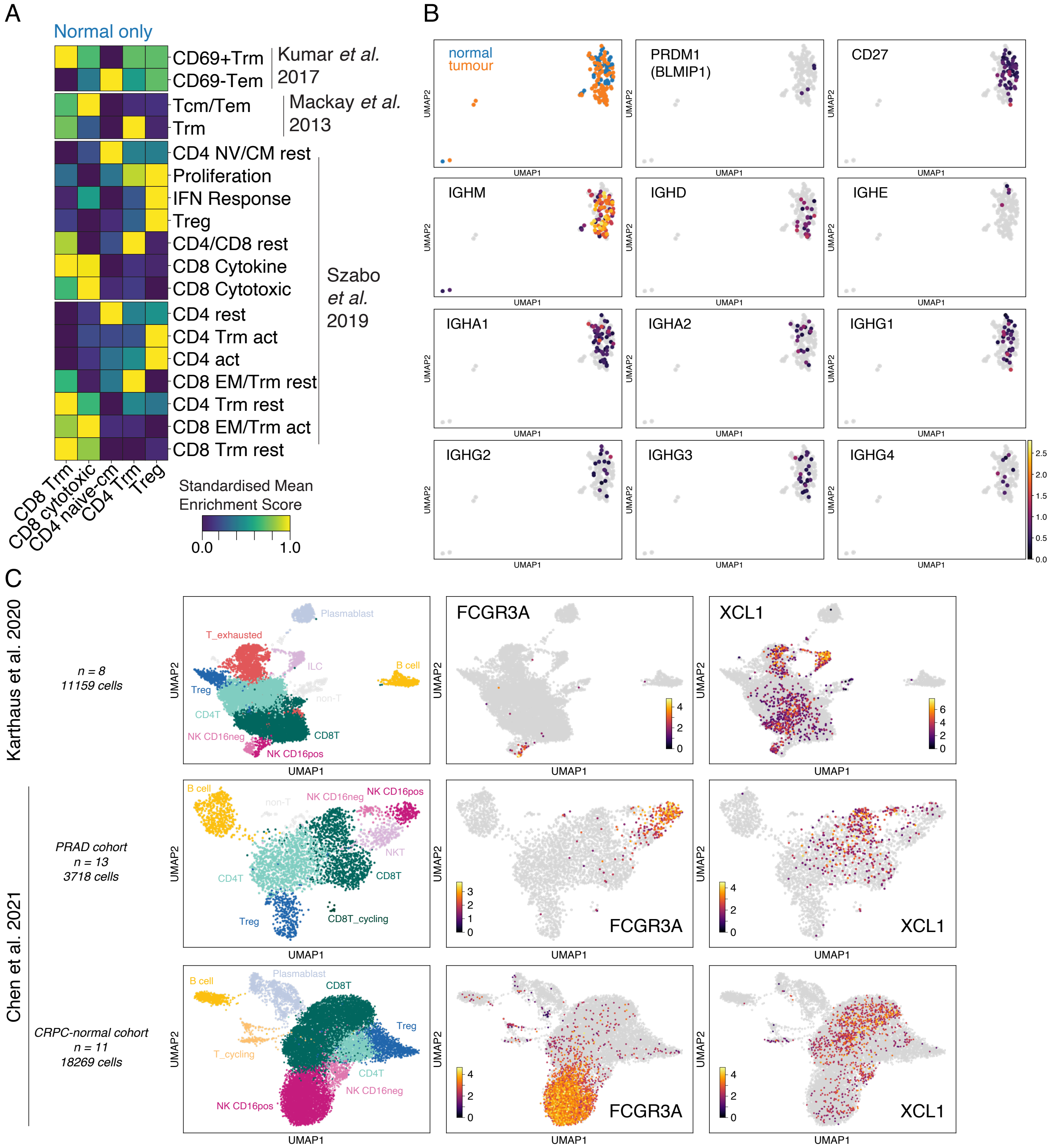
A
Normal only
CD69+Trm
CD69-Tem
Kumar et al. 2017
Tcm/Tem
Trm
Mackay et al. 2013
CD4 NV/CM rest
Proliferation
IFN Response
Treg
CD4/CD8 rest
CD8 Cytokine
CD8 Cytotoxic
CD4 rest
CD4 Trm act
CD4 act
CD8 EM/Trm rest
CD4 Trm rest
CD8 EM/Trm act
CD8 Trm rest
Szabo et al. 2019
CD8 Trm
CD8 cytotoxic
CD4 naive-cm
CD4 Trm
Treg
Standardised Mean Enrichment Score
0.0
1.0
B
normal
tumour
PRDM1 (BLMIP1)
CD27
IGHM
IGHD
IGHE
IGHA1
IGHA2
IGHG1
IGHG2
IGHG3
IGHG4
UMAP1
UMAP2
C
Karthaus et al. 2020
n = 8
11159 cells
Plasmablast
T_exhausted
ILC
B cell
Treg
non-T
CD4T
CD8T
NK CD16neg
NK CD16pos
FCGR3A
XCL1
Chen et al. 2021
PRAD cohort
n = 13
3718 cells
NKT
CD8T_cycling
CRPC-normal cohort
n = 11
18269 cells
T_cycling

Fig. S6

Fig. S6. Lymphoid cells in prosrate and comparisons to other, related to Fig. 4. (A) Heatmap of mean gene module scores using gene lists from (Mackay et al., 2013, Kumar et al., 2017, Szabo et al., 2019) in normal T cell clusters. Row enrichment value is scaled from 0 to 1 and presented as an increasing gradient from purple, blue, green to yellow which corresponds to increasing enrichment score. (B) UMAP expression plot of B cell markers in prostate B cells. Increasing colour gradient from grey (no expression), purple, orange to yellow corresponds to increasing expression value. (C) (Left) UMAP of reannotated lymphoid cells from previously published prostate cancer single-cell data datasets (Chen et al., 2021, Karthaus et al., 2020). (Right) Marker gene expression of CD16pos/neg NK population in the datasets. Grey indicates no expression.

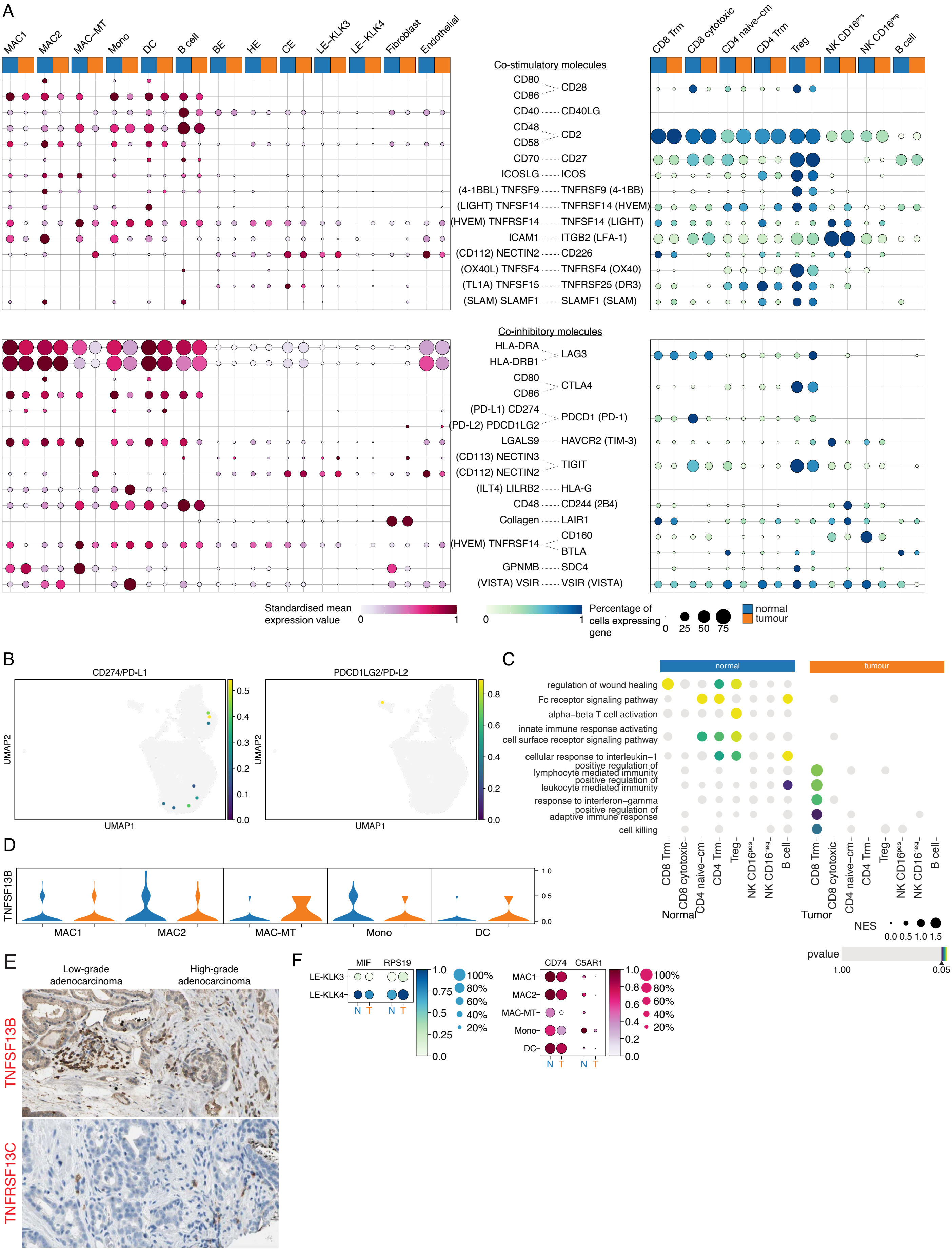
A
MAC1
MAC2
MAC-MT
Mono
DC
B cell
BE
HE
CE
LE-KLK3
LE-KLK4
Fibroblast
Endothelial
CD8 Trm
CD8 cytotoxic
CD4 naive-cm
CD4 Trm
Treg
NK CD16pos
NK CD16neg
B cell
Co-stimulatory molecules
CD80
CD86
CD28
CD40
CD40LG
CD48
CD58
CD2
CD70
CD27
ICOSLG
ICOS
(4-1BBL) TNFSF9
TNFRSF9 (4-1BB)
(LIGHT) TNFSF14
TNFRSF14 (HVEM)
(HVEM) TNFRSF14
TNFSF14 (LIGHT)
ICAM1
ITGB2 (LFA-1)
(CD112) NECTIN2
CD226
(OX40L) TNFSF4
TNFRSF4 (OX40)
(TL1A) TNFSF15
TNFRSF25 (DR3)
(SLAM) SLAMF1
SLAMF1 (SLAM)
Co-inhibitory molecules
HLA-DRA
HLA-DRB1
LAG3
CD80
CD86
CTLA4
(PD-L1) CD274
(PD-L2) PDCD1LG2
PDCD1 (PD-1)
LGALS9
HAVCR2 (TIM-3)
(CD113) NECTIN3
(CD112) NECTIN2
TIGIT
(ILT4) LILRB2
HLA-G
CD48
CD244 (2B4)
Collagen
LAIR1
(HVEM) TNFRSF14
CD160
BTLA
GPNMB
SDC4
(VISTA) VSIR
VSIR (VISTA)
Standardised mean expression value
0
1
0
1
Percentage of cells expressing gene
0
25
50
75
normal
tumour
B
CD274/PD-L1
PDCD1LG2/PD-L2
UMAP1
UMAP2
C
normal
tumour
regulation of wound healing
Fc receptor signaling pathway
alpha–beta T cell activation
innate immune response activating cell surface receptor signaling pathway
cellular response to interleukin−1
positive regulation of lymphocyte mediated immunity
positive regulation of leukocyte mediated immunity
response to interferon−gamma
positive regulation of adaptive immune response
cell killing
Normal
Tumor
NES
0.0 0.5 1.0 1.5
pvalue
1.00
0.05
D
TNFSF13B
MAC1
MAC2
MAC-MT
Mono
DC
E
Low-grade adenocarcinoma
High-grade adenocarcinoma
TNFSF13B
TNFRSF13C
F
MIF
RPS19
LE-KLK3
LE-KLK4
N T N T
CD74
C5AR1
MAC1
MAC2
MAC-MT
Mono
DC
100%
80%
60%
40%
20%

Fig. S7

Fig. S7. Expression of costimulatory and co-inhibitory molecules in prostate immune cells, related to Fig. 5. (A) Mean expression dot plot of (top) costimulatory and (bottom) coinhibitory molecules in myeloid clusters and lymphocyte clusters. Increasing expression corresponds with increasing gradient from white to red (MNP/B cell/epithelial/stromal) or white to blue (T/NK/B cell) corresponding to increasing expression value. Size of circles indicate the percentage of cells expression the gene. (B) UMAP expression plot of PD-L1 and PD-L2 in non-immune cells. Increasing colour gradient from grey (no expression), purple, blue, green to yellow corresponds to increasing expression value. (C) GSEA of GO terms in lymphoid clusters split by tumour and normal. Immune related terms are shown. Size of circles indicate normalised enrichment score (NES) and colours indicate if terms w ere significant in the clusters, which range from grey (not significant), purple, blue, green to yellow for decreasing p values. (D) Violin plots of TNFSF13B expression in myeloid cluster split by groups (N = normal; T = tumour). Expression value is standardised to a range from 0 to 1. No statistically significant comparisons observed. (E) Immunohistochemistry images of TNFSF13B and TNFRSF13C in prostate tissue. Images are sourced from the Human protein atlas (https://www.proteinatlas.org). (F) Mean expression dot plot of MIF and RPS19 in LE clusters and CD74 and C5AR1 in myeloid clusters. Increasing expression corresponds with increasing gradient from white to blue (LE clusters) or white to red (myeloid clusters). Size of circles indicate the percentage of cells expression the gene.

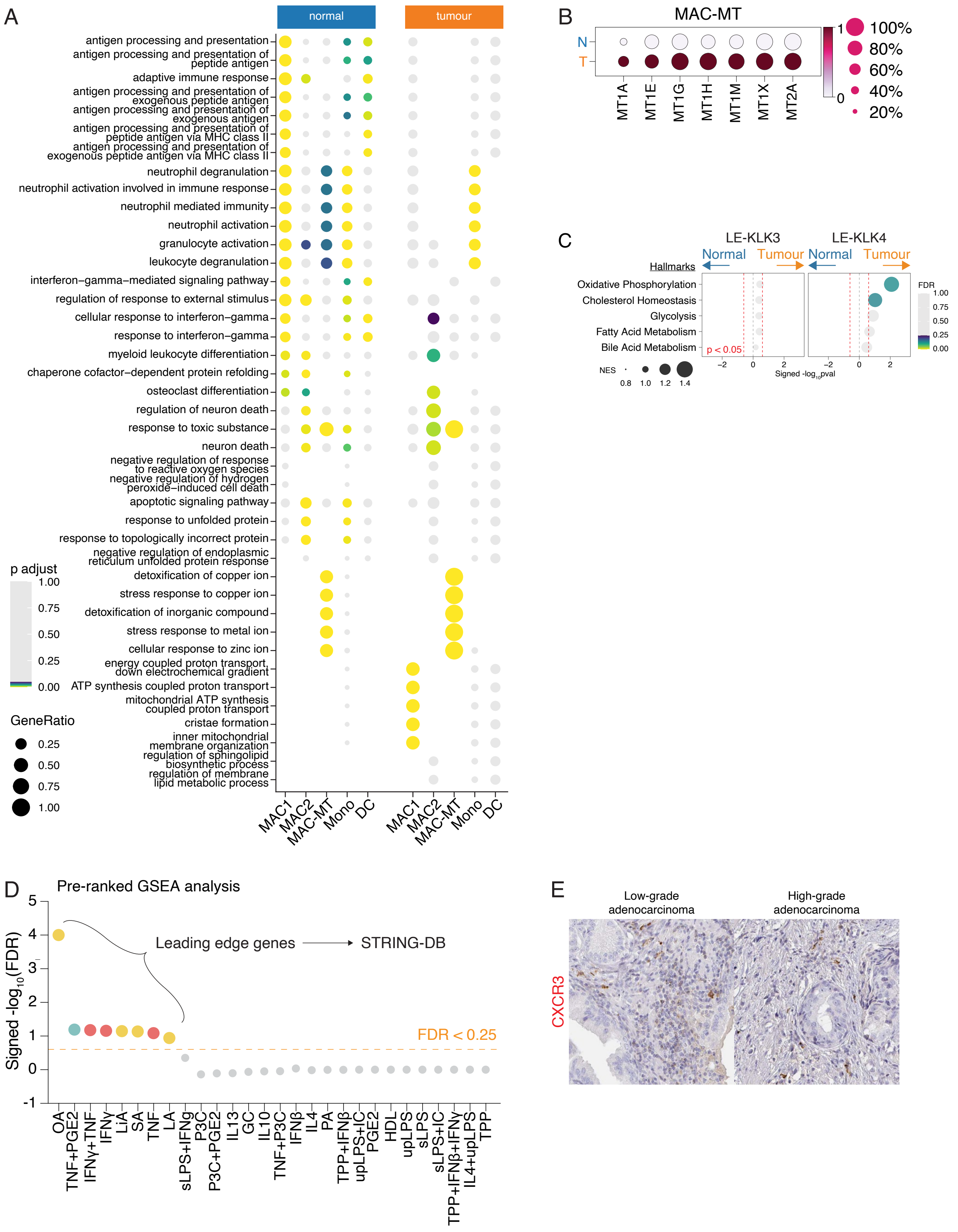
A
normal
tumour
antigen processing and presentation
antigen processing and presentation of peptide antigen
adaptive immune response
antigen processing and presentation of exogenous peptide antigen
antigen processing and presentation of exogenous antigen
antigen processing and presentation of peptide antigen via MHC class II
antigen processing and presentation of exogenous peptide antigen via MHC class II
neutrophil degranulation
neutrophil activation involved in immune response
neutrophil mediated immunity
neutrophil activation
granulocyte activation
leukocyte degranulation
interferon−gamma−mediated signaling pathway
regulation of response to external stimulus
cellular response to interferon−gamma
response to interferon−gamma
myeloid leukocyte differentiation
chaperone cofactor−dependent protein refolding
osteoclast differentiation
regulation of neuron death
response to toxic substance
neuron death
negative regulation of response to reactive oxygen species
negative regulation of hydrogen peroxide−induced cell death
apoptotic signaling pathway
response to unfolded protein
response to topologically incorrect protein
negative regulation of endoplasmic reticulum unfolded protein response
detoxification of copper ion
stress response to copper ion
detoxification of inorganic compound
stress response to metal ion
cellular response to zinc ion
energy coupled proton transport, down electrochemical gradient
ATP synthesis coupled proton transport
mitochondrial ATP synthesis coupled proton transport
cristae formation
inner mitochondrial membrane organization
regulation of sphingolipid biosynthetic process
regulation of membrane lipid metabolic process
MAC1 MAC2 MAC-MT Mono DC
MAC1 MAC2 MAC-MT Mono DC
p adjust
1.00
0.75
0.50
0.25
0.00
GeneRatio
0.25
0.50
0.75
1.00
B
MAC-MT
N
T
MT1A MT1E MT1G MT1H MT1M MT1X MT2A
1
0
100%
80%
60%
40%
20%
C
LE-KLK3
LE-KLK4
Normal Tumour Normal Tumour
Hallmarks
Oxidative Phosphorylation
Cholesterol Homeostasis
Glycolysis
Fatty Acid Metabolism
Bile Acid Metabolism
p < 0.05
−2 0 2 −2 0 2
Signed -log10pval
NES
0.8 1.0 1.2 1.4
FDR
1.00
0.75
0.50
0.25
0.00
D
Pre-ranked GSEA analysis
Leading edge genes → STRING-DB
FDR < 0.25
Signed -log10(FDR)
5 4 3 2 1 0 -1
OA TNF+PGE2 IFNγ+TNF IFNγ LiA SA TNF LA sLPS+IFNg P3C P3C+PGE2 IL13 GC IL10 TNF+P3C IFNβ IL4 PA TPP+IFNβ upLPS+IC PGE2 HDL upLPS sLPS sLPS+IC TPP+IFNβ+IFNγ IL4+upLPS TPP
E
Low-grade adenocarcinoma
High-grade adenocarcinoma
CXCR3

Fig. S8

Fig. S8. Pathway analysis of prostate myeloid cells, related to Fig. 6. (A) Gene ontology over-representation analysis of significant DEG markers in myeloid clusters split by tumour and normal. Top 5 terms for each cluster are shown. Size of circles indicate number of genes enriching pathway and colours indicate the significance which range from purple, blue, green to yellow for decreasing p values. Grey dots did not attain statistical significance. (B) Mean expression dot plot of metallothionein genes in MAC-MT cluster separated by normal or tumour in rows. Size of circle indicates the percentage of cells expressing the genes and colour indicates which group (N = normal; T = tumour) expresses higher (dark red) levels of the genes. (C) GSEA of Hallmark metabolic gene sets and in tumour vs normal for LE-KLK3 and LE-KLK4. Pathways were considered statistically significant if p value < 0.05 (marked by vertical dashed red line). Size of circles indicate normalised enrichment score (NES) and colours indicate if pathways achieved FDR < 0.25 starting from purple, blue, green to yellow as significance values decreases. (D) GSEA of macrophage stimulation signatures in MAC-MT vs other myeloid clusters. The leading edge genes from the top enriched pathways (FDR < 0.25) were selected for String-DB analysis. The colours of the circle indicates the grouping of the stimulation (red = inflammatory; yellow = fatty acid; green = mixed). (E) Immunohistochemistry images of CXCR3 in prostate tissue. Images are sourced from the Human protein atlas (https://www.proteinatlas.org).

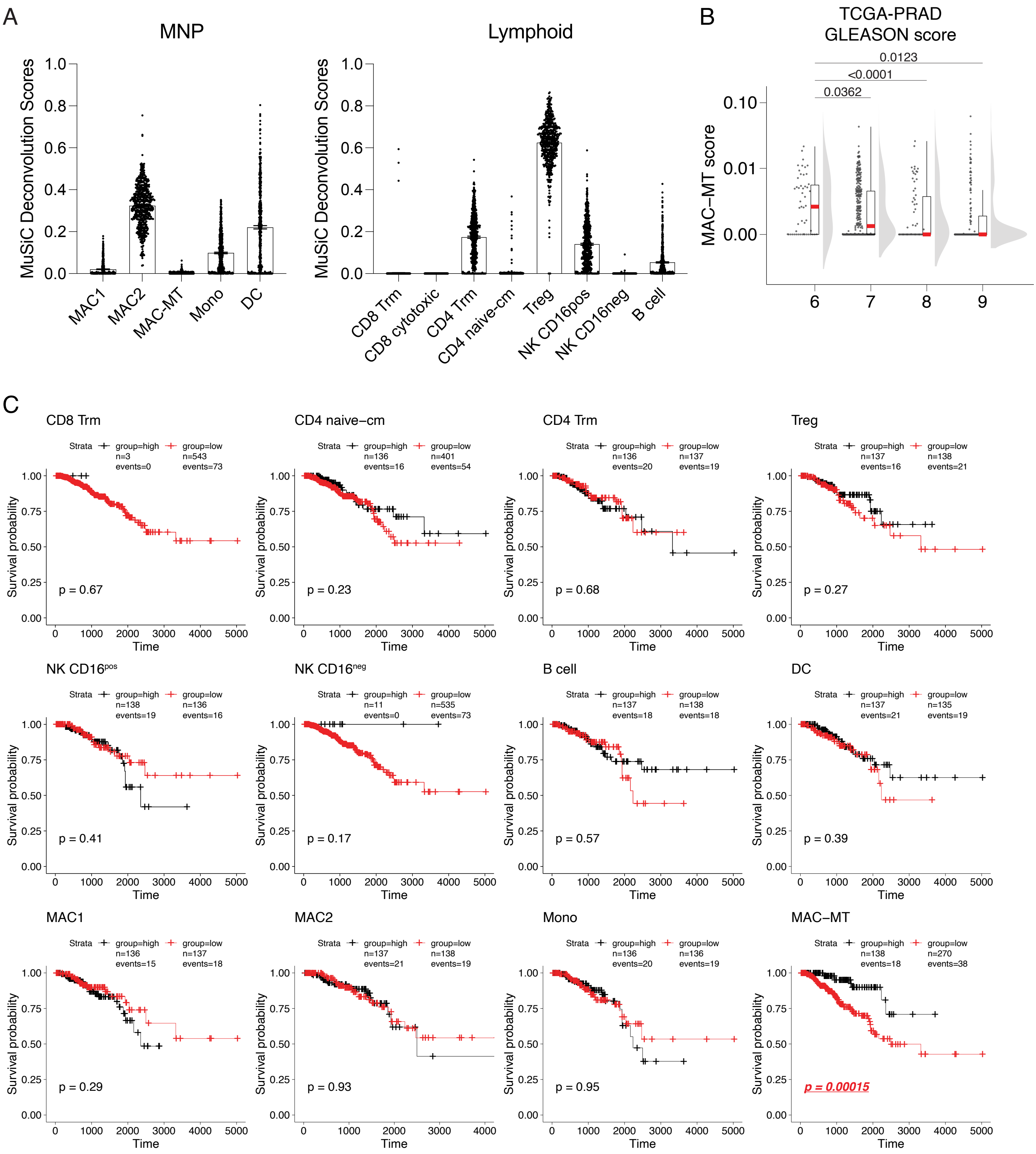

A
MNP
Lymphoid
MuSiC Deconvolution Scores
MAC1
MAC2
MAC-MT
Mono
DC
CD8 Trm
CD8 cytotoxic
CD4 Trm
CD4 naive-cm
Treg
NK CD16pos
NK CD16neg
B cell
B
TCGA-PRAD
GLEASON score
0.0123
<0.0001
0.0362
MAC–MT score
C
CD8 Trm
p = 0.67
CD4 naive–cm
p = 0.23
CD4 Trm
p = 0.68
Treg
p = 0.27
NK CD16pos
p = 0.41
NK CD16neg
p = 0.17
B cell
p = 0.57
DC
p = 0.39
MAC1
p = 0.29
MAC2
p = 0.93
Mono
p = 0.95
MAC–MT
p = 0.00015
Survival probability
Time

Fig. S9

Fig. S9. Deconvolution of immune cells in TCGA data, related to Fig. 6. (A) MuSiC deconvolution results of immune cells enrichment in TCGA-PRAD samples (n=551 samples). (B) Combined scatter-, box-, violin-plot of deconvolved MAC-MT enrichment score in TCGA data set separated by sum GLEASON scores. Kruskal-Wallis test was performed between the groups and $p<0.05$ was considered statistically significant. (C) Kaplan-Meier survival curve for TCGA-PRAD disease free index with deconvolved scores for various immune cell clusters. TCGA-PRAD samples were categorised into high (black, top 25%) and low (red, bottom 25%) of deconvolved score. Statistical analysis was performed with log rank test and $p < 0.05$ was considered statistically significant. Samples were categorised into high (black, top 25%) and low (red, bottom 25%) of deconvolved score. Statistical analysis was performed with log rank test and $p < 0.05$ was considered statistically significant.
